# The disease burden attributable to 18 occupational risks in China: an analysis for the global burden of disease study 2017

**DOI:** 10.1186/s12940-020-00577-y

**Published:** 2020-02-19

**Authors:** Jie Li, Peng Yin, Haidong Wang, Xinying Zeng, Xiao Zhang, Lijun Wang, Jiangmei Liu, Yunning Liu, Jinling You, Zhenping Zhao, Shicheng Yu, Maigeng Zhou

**Affiliations:** 1 National Center for Chronic and Noncommunicable Disease Control and Prevention, Chinese Center for Disease Control and Prevention, 27 Nanwei Road, Xicheng District, Beijing, 100050 China; 2 Institute for Health Metrics and Evaluation, University of Washington, Seattle, WA USA; 3 Chinese Center for Disease Control and Prevention, Beijing, China

**Keywords:** Death, Attributable burden, Occupational risk, Risk exposure

## Abstract

**Background:**

China has more than 18% of the global population and over 770 million workers. However, the burden of disease attributable to occupational risks is unavailable in China. We aimed to estimate the burden of disease attributable to occupational exposures at provincial levels from 1990 to 2017.

**Methods:**

We estimated the summary exposure values (SEVs), deaths and disability-adjusted life years (DALYs) attributable to occupational risk factors in China from 1990 to 2017, based on Global Burden of Disease Study (GBD) 2017. There were 18 occupational risks, 22 related causes, and 35 risk-outcome pairs included in this study. Meanwhile, we compared age-standardized death rates attributable to occupational risk factors in provinces of China by socio-demographic index (SDI).

**Results:**

The SEVs of most occupational risks increased from 1990 to 2017. There were 323,833 (95% UI 283,780 - 369,061) deaths and 14,060,210 (12,022,974 - 16,125,763) DALYs attributable to total occupational risks in China, which were 27.9 and 22.1% of corresponding global levels, respectively. For attributable deaths, major risks came from occupational particulate matter, gases, and fumes (PGFs), and for the attributable DALYs, from occupational injuries. The attributable burden was higher in males than in females. Compared with high SDI provinces, low SDI provinces, especially Western China, had higher death rates attributable to total occupational risks, occupational PGFs, and occupational injuries.

**Conclusion:**

Occupational risks contribute to a huge disease burden in China. The attributable burden is higher in males, and in less developed provinces of Western China, reflecting differences in risk exposure, socioeconomic conditions, and type of jobs. Our study highlights the need for further research and focused policy interventions on the health of workers especially for less developed provinces in China to reduce occupational health losses effectively.

## Introduction

China is the most populous country in the world with a population of 1.37 billion [1]. Over the past few decades, China has emerged as a global leader in manufacturing with growing competitiveness and increasing impact on the global economy. However, rapid economic growth also brings to the workplace a variety of risks that threaten the health of workers. There are more than 770 million workers in China, and more than 200 million workers are exposed to occupational hazards [1, 2]. It has become a priority for China to meet the challenges in the monitoring of the health of workers and in the improvement of occupational health services.

Occupational risks, as part of environmental hazards, contribute to the development of many diseases and injuries [3–9]. By evaluating burden attributable to occupational risks, accurate and comprehensive data can be offered to policymakers to effectively prevent related health losses. Although attempts have been made to estimate the burden of air pollution at the national level [10, 11], only a few studies estimated the burden of occupational carcinogens and injuries and they were limited to several provinces of China [12–14]. Additionally, occupational exposure exhibits spatial and temporal heterogeneity and is closely related to socioeconomic levels in different regions. Therefore, a comprehensive study on the spatiotemporal trend of the burden of disease attributable to occupational risks is urgently needed in China.

In this paper, we evaluated the disease burden levels attributable to 18 occupational risks and their geographical heterogeneity by socio-demographic index (SDI) in China from 1990 to 2017, as part of the Global Burden of Disease Study 2017 (GBD 2017). We aimed to find out the key problems in occupational health so as to provide useful information for occupational protection strategies and interventions in China.

## Methods

### Overview

The comparative risk assessment (CRA) approach was developed to estimate levels and trends of sex-specific, risk-specific, and cause-specific mortality and disease burden of behavioral, environmental, occupational, and metabolic risks from 1990 to 2017 for 195 countries and territories in GBD 2017 [15]. The detailed framework and data analysis methods have been provided previously [16–18]. In the CRA framework, the attributable burden was calculated as the reduction in the current disease burden if the past population exposure shifted to the counterfactual level of risk exposure. By using a consistent approach, CRA allows rankings and comparisons among deaths and DALYs attributable to various risk factors, providing further data guidance for policymakers. SDI as a combined indicator was estimated based on fertility among women, years of education and income per person. The SDI of China in 2017 was estimated in our previous article [19]. Here we focused on accessible data of occupational risks from GBD 2017 to estimate the disease burden attributable to occupational exposure in China.

### Risk factors and related causes

Risk-outcome pairs satisfying the World Cancer Research Fund (WCRF) grades of convincing or probable evidence with biologically plausible associations were included in GBD 2017 [15]. There are 18 occupational risks, 22 related causes and 35 risk-outcome pairs included in this study. The occupational risk factors hierarchy and related causes were shown in Table 1. There are six risk categories including carcinogens, asthmagens, PGFs, noise, injuries, and ergonomic factors for occupational risks. The occupational carcinogens include 13 agents classified as Group 1 carcinogens by the International Agency for Research on Cancer (IARC). The exposure definitions, International Classification of Diseases (ICD) codes of related cases, and epidemiological evidence supporting risk-outcome pairs are listed in Tables S1–S3.

**Table 1:** Occupational risk factors hierarchy and related causes included in GBD 2017

Occupational risk factors		Related causes
3	Asthmagens	Asthma
3	Carcinogens	
4	Arsenic	Tracheal, bronchus, and lung cancer
4	Asbestos	Larynx cancer
		Mesothelioma
		Ovarian cancer
		Pneumoconiosis
		Tracheal, bronchus, and lung cancer
4	Benzene	Leukemia
4	Beryllium	Tracheal, bronchus, and lung cancer
4	Cadmium	Tracheal, bronchus, and lung cancer
4	Chromium	Tracheal, bronchus, and lung cancer
4	Diesel engine exhaust	Tracheal, bronchus, and lung cancer
4	Formaldehyde	Leukemia
		Nasopharynx cancer
4	Nickel	Tracheal, bronchus, and lung cancer
4	Polycyclic aromatic hydrocarbons	Tracheal, bronchus, and lung cancer
4	Silica	Pneumoconiosis
		Tracheal, bronchus, and lung cancer
4	Sulfuric acid	Larynx cancer
4	Trichloroethylene	Kidney cancer
3	Ergonomic factors	Low back pain
3	Injuries	Road injuries
		Falls
		Drowning
		Exposure to mechanical forces
		Poisonings
		Other transport injuries
		Other unintentional injuries
		Fire, heat, and hot substances
		Animal contact
		Foreign body
3	Noise	Age-related and other hearing loss
3	Particulate matter, gases, and fumes	COPD
		Pneumoconiosis

### Estimation of exposure

Data for occupational risk factors were collected from all accessible resources. The data included raw data on Chinese economic activity proportions, occupation proportions, employment to population ratio estimates, and fatal injury rates from the International Labour Organization, survey data including China National Population Census, China Intercensal Population Sample Survey of One-Percent, China International Social Survey Programme [15]. The Spatio-temporal Gaussian process regression (ST-GPR) approach was used to integrate multiple data inputs and generate year-specific, and location-specific estimates. For each occupational risk, the theoretical minimum risk exposure level (TMREL) was assumed to no given risk exposure or the lowest levels of risk exposure without established risk-outcome (Table S1). Education, geological information and the socio-demographic level were included in models as covariates. The estimates differed for (1) occupational carcinogens, occupational noise, and occupational particulates, (2) occupational ergonomic factors and occupational asthmagens, and (3) occupational injuries using the following equations:

1 $$ {\mathrm{E}}_{r,p,l,y,s,a}=\sum \limits_e\left({\mathrm{P}}_{e,p,y}\ast {\mathrm{E}\mathrm{AP}}_{p,y,s,a}\ast {\mathrm{E}\mathrm{xposure}\ \mathrm{rate}}_{r,l,e}\right) $$

2 $$ {\mathrm{E}}_{r,p,y,s,a}=\sum \limits_e\left({P}_{occ,p,y}\ast {EAP}_{p,y,s,a}\right) $$

3 $$ \mathrm{Occupational}\ {\mathrm{fatal}\ \mathrm{injuries}}_{p,y,s,a}=\sum \limits_e\left({Injury\ rate}_{e,p,y,s}\ast {Population}_{p,y,s,a}\ast {EAP}_{p,y,s,a}\ast {P}_{e,p,y}\right) $$

Where E_r,p,l,y,s,a_ is the prevalence of exposure for risk factor r in province p at level l in year y, sex s, and age group a. P_e,p,y_ is the proportion of economically active population in province p, economic activity e, and year y. EAP_p,y,s,a_ is economically active population in province p, year y, sex s and age group a. Exposure rate_r,l,e_ is the rate of exposure to risk factor r at level l in economic activity e. E_r,p,y,s,a_ is the prevalence of exposure for risk r in province p, year y, sex s, and age group a. P_occ,p,y_ is the proportion of economically active population in occupation occ in province p, and year y. Occupational fatal injuries_p,y,s,a_ is the occupational fatal injuries counts in province p, year y, sex s and age group a. Injury rate_e,p,y,s_ is the injury rate of economic activity e in province p, year y and sex s. Population_p,y,s,a_ is the population in year y, province p, sex s and age group a. All occupational risk exposures were estimated for ages 15 and older. The estimates were further divided by the sum of all the estimates to be rescaled to sum as 1 across different categories.

### Relative risks and the population attributable fraction

Information from the cohort, pooled cohort, and case-control studies was obtained to determine the relative risk for each risk-outcome pair by systematic reviews in GBD 2017 [15]. The risk factors were categorized based on the measurement of exposure: dichotomous, polytomous, and continuous. The relative risks for each exposure category were listed in Tables S4 and S5.

The population attributable fraction (PAF) is the proportion of outcomes or causes in the population that are attributable to the associated risk factor [20]. It is estimated independently by relative risks and calculated as the proportion of the decreased outcome among a given population if the past exposure levels of risk were reduced to the counterfactual level of the TMREL in a given year.

The equation for calculating PAFs of occupational risks with the exception of injuries:

4 $$ {PAF}_{r,c,p,y,s,a}=\frac{\sum_{x=l}^u{RR}_{r,c,s,a}(x)\ast {P}_{r,p,y,s,a}(x)-{RR}_{r,c,p,s,a}\left({TMREL}_{r,s,a}\right)}{\sum_{x=l}^u{RR}_{r,c,s,a}(x)\ast {P}_{r,p,y,s,a}(x)} $$

Where PAF_r,c,p,y,s,a_ is the population attributable fraction for cause c due to risk factor r in province p, year y, sex s and age group a. RR_r,c,s,a_ is the relative risk as a function of exposure level x (ranged from lowest exposure level (l) to highest exposure level (u)) for risk r, cause c, sex s, and age group a. P_r,p,y,s,a_(x) is the distribution of exposure for risk r, in province p, year y, sex s, and age group a. TMREL_r,s,a_ is the theoretical minimum risk exposure level for risk factor r, sex s, and age group a.

The equation for calculating PAFs of occupational injuries:

5 $$ {PAF}_{p,y,s,a}=\frac{Occupational\ {fatal\ injuries}_{p,y,s,a}- TMREL}{Fatal\ \mathrm{i}{njuries}_{p,y,s,a}} $$

Where PAF_p,y,a,s_ is the population attributable fraction in province p, year y, sex s and age group a. Occupational fatal injuries_p,y,s,a_ is the occupational fatal injuries counts in province p, year y, sex s and age group a. Fatal injuries_p,y,s,a_ is the total fatal injuries counts in province p, year y, sex s and age group a, which were obtained from causes of death in GBD 2017 [15]. And the PAFs of multiple risks are aggregated by a mediation adjustment to calculate the excess attenuated risk. The PAFs for different causes were shown in Table S6.

### Estimation of attributable burden

For the given exposure risk-outcome pair, the attributable deaths were estimated as total deaths for the outcome multiplied by the PAF for the risk-outcome pair [15]. The other three metrics of burden including years of life lost (YLLs), years lived with disability (YLDs), and DALYs (the sum of YLLs and YLDs) were also assessed in a similar way. The attributable burden was estimated by location, age, sex, and year. The standard population from WHO was used to calculate age-standardized deaths and DALYs per capita for each country.

### Summary exposure values

Summary exposure values (SEV) is an exposure metric of the risk-weighted prevalence of each risk [15]. SEV standardizes the prevalence by relative risks of related causes to offer a concise comparable summary of risk exposure for different locations and years. The range of SEV is from 0 to 100%, where 0% indicates no given risk exposure in a population, and 100% means the total population is exposed to the maximum possible level for a given risk.

## Results

There were SEVs for 17 occupational risks with the exception of occupational injuries in GBD 2017 (Table 2). The leading exposure category for SEVs was ergonomic factors, followed by asthmagens, noise, and PGFs. All-age SEVs of 15 in 17 occupational risks increased from 1990 to 2017 in China. Age-standardized SEVs for three risks increased by more than 20% from 1990 to 2017: occupational exposure to benzene, trichloroethylene, and chromium. Conversely, SEVs for occupational ergonomic factors and asthmagens decreased by more than 20%.

**Table 2:** All-age and age-standardized summary exposure values for occupational risk factors, 1990, and 2017 in China, with mean percentage change for 1990–2017

Occupational risks	All-age SEVs			Age-standardized SEVs		
	1990	2017	Percentage change (%)	1990	2017	Percentage change (%)
Asthmagens	14.18 (11.46–17.52)	13.35 (11.15–15.77)	−5.84(−19.48–10.57)	14.15 (11.43–17.33)	10.77 (9.04–12.74)	−23.89(−34.57--12.36)
Ergonomic factors	21.04 (19.22–22.91)	16.62 (14.42–19.04)	−20.99(−32.18--9.47)	20.97 (19.21–22.75)	13.40 (11.66–15.30)	−36.10(−44.96--26.78)
Carcinogens						
Arsenic	0.32 (0.13–0.55)	0.55 (0.22–0.92)	67.96 (54.02–83.30)	0.35 (0.14–0.59)	0.40 (0.16–0.67)	12.88 (3.53–23.23)
Asbestos	0.30 (0.22–0.43)	0.70 (0.62–0.76)	131.64 (52.45–229.15)	0.43 (0.32–0.59)	0.51 (0.45–0.56)	20.10(−19.30–64.12)
Benzene	0.50 (0.31–0.88)	0.67 (0.40–1.19)	34.52 (27.40–43.40)	0.46 (0.28–0.82)	0.57 (0.34–1.02)	22.65 (16.16–30.74)
Beryllium	0.09 (0.08–0.09)	0.13 (0.13–0.13)	51.82 (46.12–57.95)	0.09 (0.09–0.09)	0.09 (0.09–0.10)	1.80(−1.99–5.95)
Cadmium	0.17 (0.16–0.18)	0.28 (0.26–0.31)	67.92 (55.20–81.64)	0.18 (0.18–0.19)	0.21 (0.19–0.22)	12.83 (4.22–22.04)
Chromium	0.34 (0.32–0.35)	0.60 (0.55–0.65)	78.75 (63.86–94.15)	0.36 (0.35–0.38)	0.43 (0.40–0.47)	20.18 (10.22–30.54)
DEE	1.41 (1.36–1.46)	2.41 (2.28–2.55)	71.01 (60.03–82.29)	1.52 (1.47–1.58)	1.75 (1.65–1.85)	14.92 (7.51–22.45)
Formaldehyde	0.78 (0.74–0.81)	1.02 (0.92–1.11)	30.80 (18.62–44.18)	0.73 (0.69–0.76)	0.87 (0.79–0.94)	19.28 (8.09–31.25)
Nickel	0.36 (0.12–0.94)	0.54 (0.18–1.42)	51.27 (40.12–64.95)	0.39 (0.13–1.01)	0.39 (0.13–1.03)	1.63(−5.88–10.79)
PAHs	0.68 (0.65–0.70)	1.19 (1.10–1.28)	76.01 (61.72–90.71)	0.73 (0.70–0.76)	0.86 (0.80–0.93)	18.33 (8.70–28.24)
Silica	3.20 (1.68–6.99)	4.49 (2.30–9.68)	40.16 (29.8–51.94)	3.45 (1.81–7.54)	3.25 (1.67–7.01)	−5.75(−12.79–2.12)
Sulfuric acid	0.76 (0.55–1.31)	1.20 (0.86–2.12)	58.34 (47.62–69.35)	0.82 (0.59–1.41)	0.87 (0.62–1.53)	6.32(−0.87–13.70)
Trichloroethylene	0.19 (0.18–0.19)	0.34 (0.32–0.36)	80.12 (69.16–93.03)	0.20 (0.20–0.21)	0.24 (0.23–0.26)	21.05 (13.63–29.70)
Noise	8.11 (7.67–8.57)	12.30 (11.75–12.95)	51.73 (46.79–57.63)	9.23 (8.73–9.83)	9.24 (8.82–9.76)	0.12(−2.85–3.28)
PGFs	8.30 (6.88–10.32)	12.12 (10.08–14.70)	46.09 (35.82–55.38)	9.28 (7.72–11.42)	9.18 (7.7–11.22)	−1.10(−4.69–2.89)

*SEV* Summary exposure value, *UI* Uncertain interval, *PGFs* Particulate matter, gases, and fumes, *DEE* Diesel engine exhaust, *PAHs* Polycyclic aromatic hydrocarbons

As shown in Table 3, there were 323,833 (95% UI 283780–369,061) deaths attributable to total occupational risks in 2017, China, which accounted for 27.9% of global attributable deaths. The deaths attributable to PGFs, carcinogens, injuries, and asthmagens accounted for 57.8, 21.1, 20.8, 0.2% of deaths attributable to total occupational risks in 2017, respectively. From 1990 to 2017, age-standardized death rate attributable to injuries, PGFs and asthmagens declined by more than 60%, but the rate attributable to carcinogens increased by 16.8%.

**Table 3:** Deaths and age-standardized death rate for occupational risk factors, 1990, and 2017 in China, with mean percentage change for 1990–2017

Occupational risks	Deaths			Age-standardized death rate (per 100,000)		
	1990	2017	Percentage change (%)	1990	2017	Percentage change (%)
Asthmagens	3039 (1765–4108)	792 (631–992)	−73.9(−82.0--49.8)	0.30 (0.17–0.42)	0.04 (0.03–0.05)	−87.1(−91.3--74.6)
Carcinogens	26,546 (20385–33,149)	68,396 (53282–85,467)	157.7 (121.5–200.4)	3.00 (2.33–3.70)	3.50 (2.75–4.35)	16.8(− 0.1–36.8)
Arsenic	1324 (514–2188)	3725 (1402–6118)	181.4 (149.0–218.6)	0.14 (0.05–0.23)	0.18 (0.07–0.29)	29.9 (15.1–47.1)
Asbestos	5796 (3962–8718)	24,264 (16700–32,287)	318.7 (185.4–451.4)	0.78 (0.54–1.17)	1.36 (0.94–1.81)	73.6 (20.0–126.9)
Benzene	380 (107–643)	372 (108–625)	− 2.22(− 15.9–23.3)	0.03 (0.01–0.05)	0.02 (0.01–0.03)	−32.0(− 42.0--15.0)
Beryllium	57 (47–68)	149 (122–179)	161.4 (132.5–188.4)	0.01 (0.00–0.01)	0.01 (0.01–0.01)	20.7 (8.2–33.0)
Cadmium	129 (109–150)	364 (302–434)	182.1 (148.9–219.8)	0.01 (0.01–0.02)	0.02 (0.01–0.02)	30.2 (15.2–47.3)
Chromium	244 (216–277)	727 (625–830)	197.5 (163.3–235.1)	0.03 (0.02–0.03)	0.03 (0.03–0.04)	37.3 (21.8–54.1)
DEE	2762 (2418–3125)	8083 (6960–9319)	192.7 (155.7–224.9)	0.28 (0.25–0.32)	0.38 (0.33–0.44)	35.1 (18.6–49.7)
Formaldehyde	408 (317–505)	391 (306–494)	− 4.2(− 20.7–14.5)	0.03 (0.03–0.04)	0.02 (0.02–0.03)	− 41.1(− 49.9--31.6)
Nickel	1488 (292–3334)	3803 (762–8450)	155.5 (126.1–188.6)	0.15 (0.03–0.34)	0.18 (0.04–0.40)	18.0 (4.4–33.1)
PAHs	852 (722–980)	2500 (2092–2937)	193.5 (157.4–231.2)	0.09 (0.07–0.10)	0.12 (0.10–0.14)	35.5 (19.4–52.8)
Silica	13,260 (8742–18,469)	25,073 (14869–35,605)	89.1 (51.3–123.9)	1.46 (0.97–2.02)	1.23 (0.74–1.73)	− 15.7(− 34.0–1.0)
Sulfuric acid	419 (173–778)	719 (301–1348)	71.8 (56.5–91.2)	0.04 (0.02–0.08)	0.03 (0.01–0.06)	− 21.3(− 28.4--12.4)
Trichloroethylene	4 (1–7)	14 (3–26)	285.4 (172.9–364.0)	0.00 (0.00–0.00)	0.00 (0.00–0.00)	81.1 (27.8–118.2)
Injuries	142,834 (120521–167,777)	67,461 (57132–79,340)	−52.8(−61.9--41.0)	11.95 (10.10–14.02)	3.84 (3.25–4.52)	− 67.9(− 74.0--59.8)
PGFs	240,075 (197593–281,136)	187,184 (148439–228,139)	−22.0(− 31.5--5.1)	37.20 (29.5–44.73)	11.36 (8.82–14.03)	− 69.5(− 72.5--63.2)
All occupational risks	412,493 (362216–461,292)	323,833 (283780–369,061)	− 21.5(− 29.5--10.8)	52.45 (44.51–60.48)	18.74 (16.17–21.56)	− 64.3(− 67.5--59.3)

*UIs* Uncertainty intervals, *PGFs* Particulate matter, gases, and fumes, *DEE* Diesel engine exhaust, *PAHs* Polycyclic aromatic hydrocarbons

As shown in Table 4, there were 14,060,210 (12,022,974 - 16,125,763) DALYs attributable to total occupational risks in 2017, China, which accounted for 22.1% of global attributable DALYs. The DALYs attributable to occupational injuries, PGFs, ergonomic factors, noise, carcinogens, and asthmagens accounted for 32.4, 28.3, 14.3, 12.4, 11.7, and 0.8% of DALYs attributable to total occupational risks in 2017, respectively. From 1990 to 2017, occupational injuries were consistently the leading risk of attributable DALYs in China. Although age-standardized DALY rate attributable to total occupational risks declined by 56.1%, occupational exposure to trichloroethylene, asbestos, chromium, polycyclic aromatic hydrocarbons (PAHs), and diesel engine exhaust (DEE) increased by more than 20%. Among all related causes, COPD was the leading cause of the attributable burden in 2017 (Table S7).

**Table 4:** DALYs and age-standardized DALY rate for occupational risk factors, 1990, and 2017 in China, with mean percentage change for 1990–2017

Occupational risks	DALYs, in thousand			Age-standardized DALY rate (per 100,000)		
	1990	2017	Percentage change (%)	1990	2017	Percentage change (%)
Asthmagens	194.5(144.5–244.7)	114.8(79.8–158.3)	− 41.0(− 54.5--20.9)	17.39(12.85–21.92)	6.28(4.34–8.68)	− 63.9(− 72.6--50.8)
Carcinogens	766.0(590.4–959.8)	1650.5(1260.4–2089.6)	115.5(87.2–147.1)	78.63(60.59–98.03)	80.49(62.23–101.39)	2.4(− 11.2–17.94)
Arsenic	40.4(15.7–66.8)	101.8(38.3–167.1)	152.2(123.1–185.8)	4.05(1.58–6.69)	4.77(1.80–7.83)	17.9(4.4–33.7)
Asbestos	124.8(85.7–190.1)	442.1(304.7–589.5)	254.3(135.1–375.6)	14.57(10.06–22.02)	22.94(15.85–30.54)	57.5(6.0–108.6)
Benzene	20.1(5.6–33.9)	17.1(5.0–28.7)	− 14.7(− 27.7–8.8)	1.56(0.44–2.65)	1.01(0.29–1.69)	− 35.5(− 45.8--17.8)
Beryllium	1.8(1.5–2.1)	4.1(3.3–4.9)	133.7(107.5–160.1)	0.18(0.15–0.21)	0.19(0.16–0.23)	9.3(− 2.3–20.5)
Cadmium	3.9(3.3–4.6)	10.0(8.3–11.9)	152.9(121.3–187.0)	0.39(0.33–0.46)	0.47(0.39–0.56)	18.2(4.4–34.0)
Chromium	7.5(6.6–8.4)	19.9(17.2–22.8)	166.8(135.6–201.4)	0.75(0.66–0.84)	0.93(0.81–1.07)	24.8(10.4–40.6)
DEE	84.3(74.2–95.5)	220.9(190.9–253.5)	161.9(129.2–191.1)	8.45(7.44–9.55)	10.35(8.97–11.87)	22.5(7.5–35.9)
Formaldehyde	19.9(15.2–24.8)	16.5(13.0–20.7)	− 17.2(− 31.4--0.3)	1.64(1.26–2.03)	0.91(0.72–1.12)	− 44.6(− 52.9--35.0)
Nickel	45.5(8.9–101.5)	104.0(20.8–231.5)	128.7(102.0–158.4)	4.56(0.90–10.19)	4.87(0.98–10.84)	7.0(− 5.4–20.8)
PAHs	26.0(22.0–29.8)	68.4(57.1–80.0)	163.1(129.3–198.0)	2.60(2.21–2.99)	3.21(2.68–3.76)	23.1(7.9–39.1)
Silica	396.2(259.5–548.7)	673.4(395.7–961.1)	70.0(37.3–99.9)	40.31(26.54–55.58)	32.12(19.09–45.62)	− 20.3(− 35.6--6.1)
Sulfuric acid	13.0(5.4–24.3)	20.9(8.8–39.0)	60.3(45.9–78.7)	1.30(0.54–2.43)	0.97(0.41–1.82)	− 25.4(− 32.2--16.9)
Trichloroethylene	0.1(0.0–0.2)	0.4(0.1–0.8)	244.5(145.3–313.0)	0.01(0.00–0.02)	0.02(0.00–0.04)	66.8(18.3–99.6)
Ergonomic factors	2245.6(1544.9–3125.8)	2010.5(1372.6–2791.7)	− 10.5(− 20.4--0.5)	196.81(135.56–275.34)	107.48(74.45–147.55)	− 45.4(− 50.4--39.5)
Injuries	8670.1(7298.9–10,281.7)	4557.6(3748.9–5420.7)	− 47.4(− 57.8--34.4)	705.89(594.31–836.67)	268.58(221.03–319.58)	− 62.0(− 69.4--52.5)
Noise	1000.0(678.0–1427.0)	1742.2(1186.1–2455.4)	74.2(69.6–79.4)	95.44(65.10–134.64)	90.92(61.88–128.17)	− 4.7(− 7.0--2.4)
PGFs	5281.4(4504.0–6043.7)	3984.6(3368.8–4617.9)	−24.6(− 30.6--12.9)	660.90(557.37–760.57)	216.42(181.99–253.00)	−67.2(−69.6--62.2)
All occupational risks	18,157.6(15,945.0–20,433.3)	14,060.2(12,023.0–16,125.8)	− 22.57(− 30.3--14.0)	1755.07(1543.63–1971.82)	770.18(662.71–885.84)	− 56.1(− 60.4--51.3)

*UIs* Uncertainty intervals, *DALYs* Disability-adjusted life year, *PGFs* Particulate matter, gases, and fumes, *DEE* Diesel engine exhaust, *PAHs* Polycyclic aromatic hydrocarbons

**Fig. 1:**
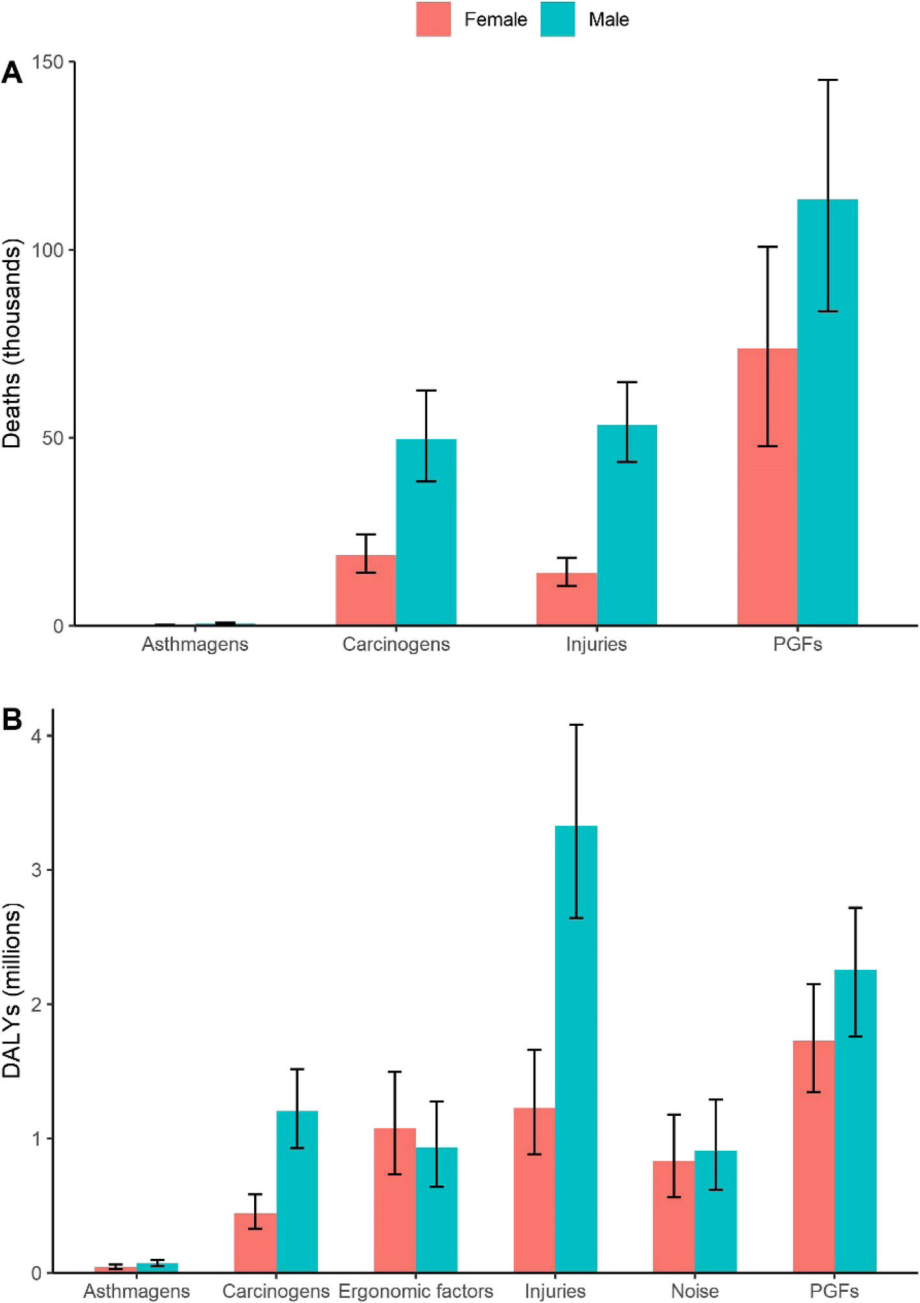
Burden attributable to occupational risks stratified by gender in China, 2017, for deaths (**a**), and DALYs (**b**). DALYs: disability-adjusted life years

For different genders, we have not observed a wide difference in SEV between males and females (Table S8). Deaths attributable to total occupational risks in males were over 2.0 times than in females in 2017. As shown in Fig. 1, the leading risk was occupational PGFs for deaths in both sexes, but for DALYs, the leading risk was occupational PGFs in females and occupational injuries in males. DALYs attributable to total occupational risks were higher in males than in females.

**Fig. 2:**
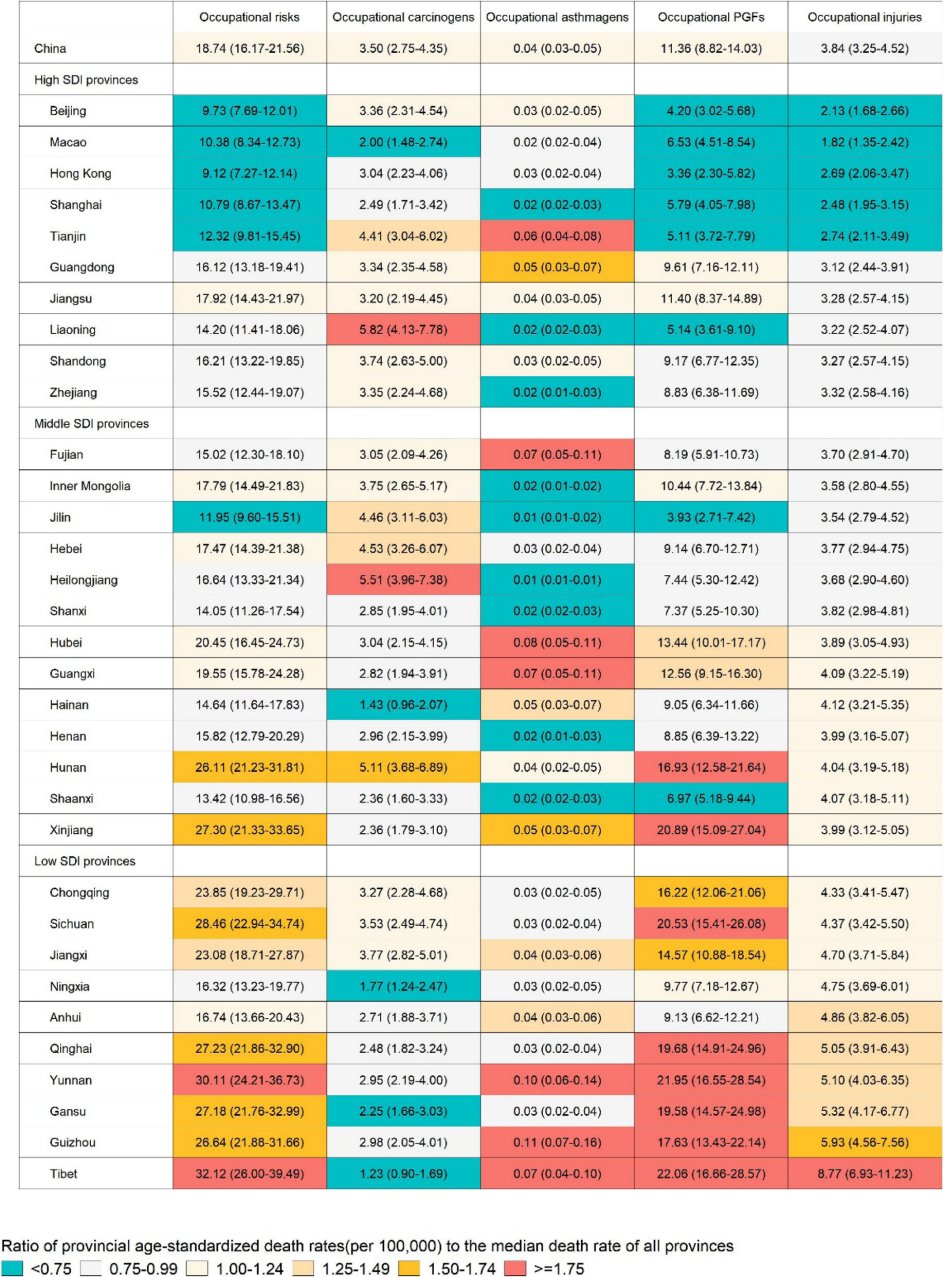
Age-standardized death rates (per 100,000) attributable to total occupational risks, occupational carcinogens, occupational asthmagens, occupational PGFs, and occupational injuries in the provinces of China grouped by SDI, 2017. SDI: socio-demographic index; PGFs: particulate matter, gases, and fumes

**Fig. 3:**
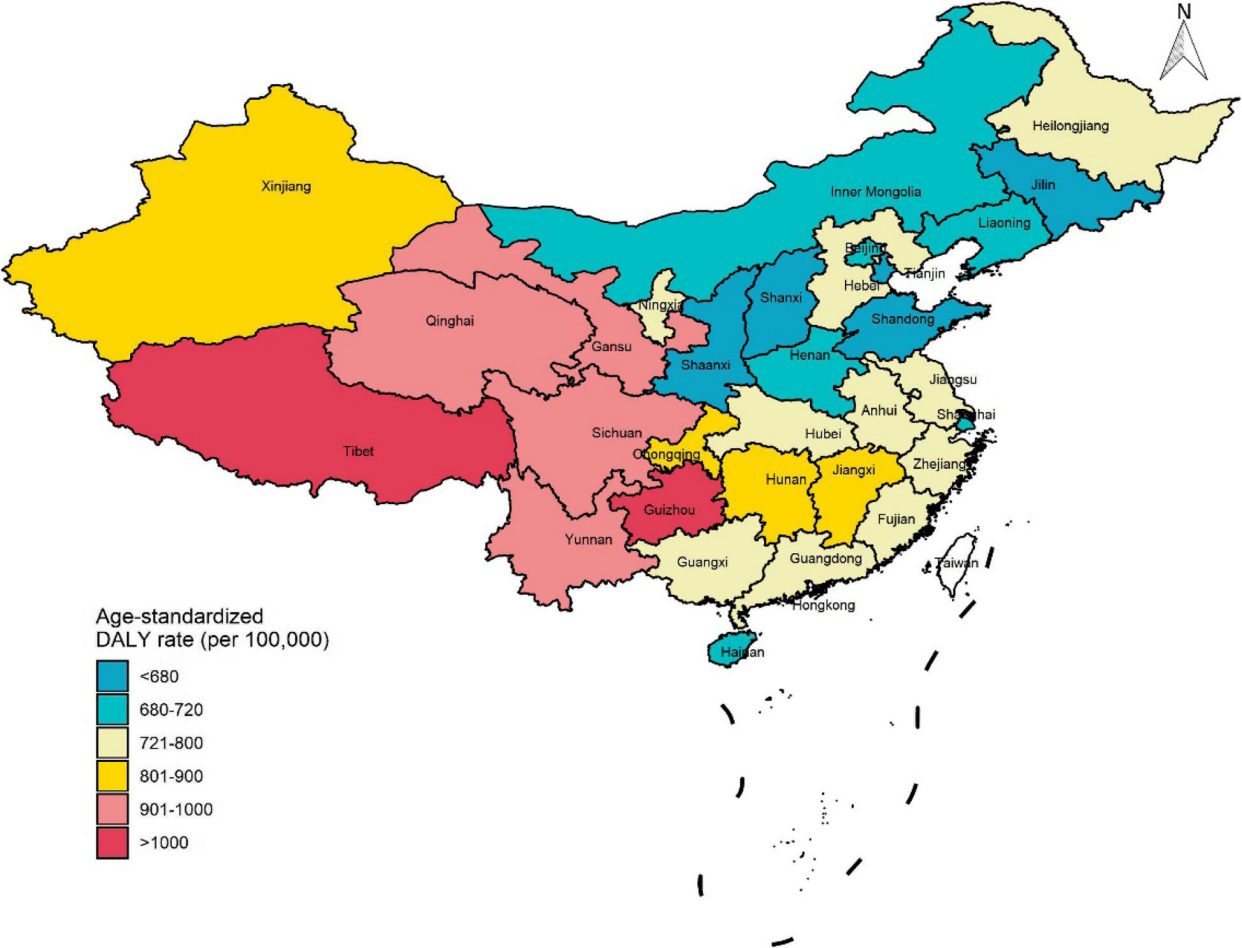
Age-standardized DALY rate (per 100,000) attributable to total occupational risks by the provinces of China, 2017. DALY: disability-adjusted life-year

Age-standardized death rates attributable to total occupational risks, carcinogens, asthmagens, PGFs, and injuries in the provinces of China grouped by SDI in 2017 are shown in Fig. 2. Compared with high SDI provinces, low SDI provinces had higher death rates attributable to total occupational risks, occupational PGFs, and occupational injuries. Western China, especially for Yunnan and Tibet, had the highest death rates attributable to total occupational risks, while Liaoning and Heilongjiang in Eastern China had the highest death rates attributable to occupational carcinogens. The highest death rates attributable to occupational PGFs and injuries mainly concentrated in Western China. Age-standardized DALY rates attributable to total occupational risks by the provinces of China in 2017 showed similar results that Western China had higher occupational age-standardized DALY rates than other regions in China (Fig. 3).

## Discussion

To our knowledge, this study presents the first comprehensive assessment so far of burden attributable to occupational risks at the provincial level of China in 2017. China has a huge labor force. The most up-to-date estimation of the disease burden attributable to occupational exposures provides important insights into health losses related to occupational risks in China. Meanwhile, the standardized assessment of attributable burden to different risk factors enables direct comparison and priority ranking.

The SEV provides a concise and comparable summary of risk exposure for different locations and years [15]. Rapid industrialization in China has been accompanied by increased occupational carcinogen exposures. Our study showed SEVs of most occupational carcinogens increased in the past 28 years, especially for asbestos, trichloroethylene. Over the past half-century, the production of asbestos in the world has been transferred to developing countries [21]. China produces about 16% of global total output and is the second-biggest consumer of asbestos in 2016 [22]. Trichloroethylene use has been increased with China’s growing telecommunications, electronic, and microelectronics industries since the early 1990s [23].

China has the largest occupational disease burden in the world. We estimated that 0.3 million deaths and 14.1 million DALYs were attributable to total occupational risks in 2017, China, which might be attributed to the huge number of labor force. Occupational injuries were the leading risk for attributable DALYs in China. A previous report showed occupational injuries and disabilities were common among Chinese workers [24]. In comparison, the leading risk for occupational burden was ergonomic factors in developed countries. Socioeconomic inequality is one of the main reasons for this difference [24–28]. The type and income of occupations, age, and education levels are important influence factors of occupational injuries [25, 26]. The economic transition from agriculture to manufacturing industry causes the migration of great rural labor forces into factories, and migrant workers have become an important part of the population who are subject to occupational hazards [29, 30]. For example, migrant workers account for 80% of total workers in the construction industry [24]. With poor educational backgrounds, migrant workers are only able to locate jobs with low wages, high-risk operation, and long working hours. They also have a lower sense of self-protection and less experience. Almost all of the occupational injuries and deaths are preventable [31]. Therefore, more vocational training and safety education targeting migrant workers are necessary to prevent health loss in China.

Although the burden attributable to all occupational risks is higher in China, it has declined more than 50% over the past three decades. The Chinese government has made numerous efforts to protect the health of workers in China, including revision of the occupational law, development of new technologies and monitoring of occupational diseases. It is further supported by coincidence of increased SEVs for occupational noises, PGFs, benzene and formaldehyde and reduced burden attributable to these risks. However, the burden attributable to occupational carcinogens, especially for asbestos, trichloroethylene, chromium, PAHs, and DEE still shows marked increases. Notably, among the 13 occupational carcinogens included in this study, only cancers caused by arsenic, asbestos, benzene, and chromium are classified into occupational cancer in the Categories and Catalogs of Occupational Diseases in China. Thus, our results provided basis for policymakers to reevaluate the potential of cancers caused by other nine carcinogens as national legal occupational disease.

The western provinces in China have the highest death and DALY rates attributable to occupational risks. The spatial inequality of disease burden for occupational hazards is similar to previous studies on environmental and occupational burden of diseases in Iran [28, 32], which might be attributed to the regional inequality of socioeconomic level and medical services of occupational diseases. Institutions certificated to diagnose the occupational disease concentrate in big cities and developed areas, with few institutions in less developed areas, especially for Western China. The imbalance between supply and demand is challenging the medical services of occupational diseases for workers from the western region.

A number of limitations exist in our estimates. The most important limitation is the lack of accessible data on the prevalence of occupational exposure in China. Scarce data are available for the specific exposure levels of occupational risks, compared with air pollution and hazards in drinking water [11, 33–35]. The exposure levels are estimated by the proportion of the population in specific types of work where the exposures are expected. The exposure assessment for occupational carcinogens was based on carcinogen exposure (CAREX) database, which included 32 million workers exposed to almost all known and suspected carcinogens in the 15 countries of the European Union in the early 1990s [36, 37]. However, workers in developing countries are likely to be exposed to higher levels of occupational hazards than developed countries. Employing CAREX data may underestimate the health effects in developing countries [38]. Meanwhile, China is a top producer of cement, coal, iron, steel with rich mineral reserves and resources. Rapid industrialization in China in recent years also brings more categories of jobs and occupational hazards. More efforts are required to more accurately determine the proportion of the working population exposed to occupational hazards in China.

Due to the stringent causal criteria, many risk-outcome pairs were hampered to be included in GBD estimates, which leads to underestimation of the disease burden attributable to occupational risks, in comparison with other published studies [39–42]. For example, benzene can cause not only leukemia but also myelodysplastic syndromes, multiple myeloma and non-Hodgkin lymphoma [43–45]. Apart from lung cancer, arsenic exposure can also cause urothelial cancer, skin cancer, and cardiovascular diseases [46]. Meanwhile, disabilities in GBD methods are associated with health conditions confined to the most common sequelae of occupational exposure but not mental disorders [42, 47]. Furthermore, there are joint effects attributable to co-exposure of multiple occupational hazards. For example, exposure to benzene interacts with work stress can reduce birth weight in petrochemical workers [48]. As most of the occupational risk-outcome pairs cannot be accessed by randomized controlled trials, evidence from epidemiological studies and toxicological studies deserves consideration in future estimates [49].

Finally, there are many uncertainties of applying the default parameters from the developed country to China. The relative risk is a critical parameter for the PAF and SEV estimations. It is derived from prospective observational studies and case-control studies by systematic reviews [15]. However, most of the relative risks come from studies conducted in high-income countries with lower occupational exposure, rather than low- and middle-income countries, which may cause biased estimates of PAF and SEV for a given population [49]. Moreover, the estimation of the TMREL is also a key step in the calculation of the PAF. A minor change of the TMREL can lead to relatively large changes in the PAF [49]. For all occupational risks, the TMREL is defined as no corresponding occupational exposure or background level to a given risk [15]. However, the background level of occupational hazards including carcinogens, asthmagens, and noises is often difficult to specify [36]. Additional epidemiological studies in regions with both high and low occupational exposure should be encouraged and supported to estimate relative risks and TMREL more accurately in China.

A human capital approach is suggested as an alternative method to provide accurate estimates of the impact of chemical exposure on population health [42, 49, 50]. It is a health economic method to assign monetary costs associated with adverse outcomes, including but not limited to traditional physical health such as cognitive deficits [42, 49]. A hybrid approach of the existing GBD method and the human capital approach should be encouraged to obtain more comprehensive and accurate estimates of the burden attributable to occupational risks.

Although the current method in GBD could not fully estimate the impacts of occupational risks on population health, the results were estimated from all accessible resources to produce relatively valid estimates up till now. Moreover, we take socioeconomic impact into our estimates to analyze differences among regions with different levels of development. Further work to increase the quality and availability of data are expected to devote adequate evidence for more valid estimates in China.

In conclusion, China is facing a tremendous disease burden attributable to occupational risk factors. Although the burden attributable to total occupational risks decreased between 1990 and 2017, the burden attributable to occupational carcinogens is rising greatly. The attributable burden is higher in males, and in less developed provinces of Western China. Our estimates will benefit policymakers to focus on preventing and reducing the health losses of workers in China.

## Supplementary information

**Additional file 1: Table S1.** Occupational risk factors hierarchy, exposure definitions, and TMREL. **Table S2**. List of ICD codes. **Table S3**. Epidemiological evidences. **Table S4**. Relative risks A. **Table S5**. Relative risks B. **Table S6**. PAF.

**Additional file 2: Table S7.** Attributable burden of all occupational risks by causes. **Table S8**. Sex-specific age-standardized SEVs of occupational risks.

## Data Availability

Please contact the author for data requests.

## Abbreviations

CAREX: Carcinogen exposure database
COPD: Chronic Obstructive Pulmonary Disease
CRA: Comparative risk assessment
DALYs: Disability-adjusted life years
DEE: Diesel engine exhaust
GBD: Global burden of disease
ICD: International Classification of Disease
PAF: Population attributable fraction
PAHs: Polycyclic aromatic hydrocarbons
PGFs: Occupational particulate matter, gases, and fumes
SDI: Socio-demographic index
SEV: Summary exposure value
ST-GPR: Spatio-temporal Gaussian process regression
TMREL: Theoretical minimum risk exposure level
UI: Uncertain interval
YLDs: Years lived with disability
YLLs: Years of life lost
