# Supplementary material for: The disease burden attributable to 18 occupational risks in China: an analysis for the global burden of disease study 2017
Source: Environ Health. 2020 Feb 19;19:21. doi: 10.1186/s12940-020-00577-y (PMC7031932; doi:10.1186/s12940-020-00577-y)
Supplement: Supplementary file 1 — Additional file 1: Table S1. Occupational risk factors hierarchy, exposure definitions, and TMREL. Table S2. List of ICD codes. Table S3. Epidemiological evidences. Table S4. Relative risks A. Table S5. Relative risks B. Table S6. PAF. [file 12940_2020_577_MOESM1_ESM.docx]

**Supporting Information**

**1. Occupational diseases burden estimation methods of GBD 2017**

The supplementary materials presented here is adapted from the following sources：

(1) GBD 2017 Global, regional, and national age-sex-specific mortality and life expectancy, 1950-2017: a systematic analysis for the Global Burden of Disease Study 2017. Lancet. 2018;392(10159):1684-1735.

(2) GBD 2017 Global, regional, and national age-sex-specific mortality for 282 causes of death in 195 countries and territories, 1980-2017: a systematic analysis for the Global Burden of Disease Study 2017. Lancet. 2018;392(10159):1736-1788.

(3) GBD 2017 Global, regional, and national incidence, prevalence, and years lived with disability for 354 diseases and injuries for 195 countries and territories, 1990–2017: a systematic analysis for the Global Burden of Disease Study 2017. Lancet. 2018; 392(10159): 1789–1858.

(4) GBD 2017 Global, regional, and national comparative risk assessment of 84 behavioral, environmental and occupational, and metabolic risks or clusters of risks for 195 countries and territories, 1990-2017: a systematic analysis for the Global Burden of Disease Study 2017. Lancet. 2018;392(10159):1923-1994.

**Table S1.** Occupational risk factors hierarchy, exposure definitions, theoretical minimum risk exposure level included in GBD 2017*

| Occupational risk factors | | Exposure definition | Theoretical minimum risk exposure level |
| --- | --- | --- | --- |
| 3 | Asthmagens | Proportion of the population currently exposed to asthmagens at work or through their occupation | Background asthmagen exposures |
| 3 | Carcinogens |  |  |
| 4 | Arsenic | Proportion of the population ever exposed to arsenic at work or through their occupation | No occupational exposure to arsenic |
| 4 | Asbestos | Proportion of the population with cumulative lifetime exposure to occupational asbestos | No occupational exposure to asbestos |
| 4 | Benzene | Proportion of the population ever exposed to benzene at work or through their occupation | No occupational exposure to benzene |
| 4 | Beryllium | Proportion of the population ever exposed to beryllium at work or through their occupation | No occupational exposure to beryllium |
| 4 | Cadmium | Proportion of the population ever exposed to cadmium at work or through their occupation | No occupational exposure to cadmium |
| 4 | Chromium | Proportion of the population ever exposed to chromium at work or through their occupation | No occupational exposure to chromium |
| 4 | Diesel engine exhaust | Proportion of the population ever exposed to diesel engine exhaust at work or through their occupation | No occupational exposure to diesel engine exhaust |
| 4 | Formaldehyde | Proportion of the population ever exposed to formaldehyde at work or through their occupation | No occupational exposure to formaldehyde |
| 4 | Nickel | Proportion of the population ever exposed to nickel at work or through their occupation | No occupational exposure to nickel |
| 4 | Polycyclic aromatic hydrocarbons | Proportion of the population ever exposed to polycyclic aromatic hydrocarbons at work or through their occupation | No occupational exposure to polycyclic aromatic hydrocarbons |
| 4 | Silica | Proportion of the population ever exposed to silica at work or through their occupation | No occupational exposure to silica |
| 4 | Sulfuric acid | Proportion of the population ever exposed to sulfuric acid at work or through their occupation | No occupational exposure to sulfuric acid |
| 4 | Trichloroethylene | Proportion of the population ever exposed to trichloroethylene at work or through their occupation | No occupational exposure to trichloroethylene |
| 3 | Ergonomic factors | Proportion of the population who are exposed to ergonomic risk factors for low back pain at work or through their occupation | All individuals have the ergonomic factors of clerical and related workers |
| 3 | Injuries | Proportion of the population at risk to injuries related to work or through their occupation | The rate of injury deaths per 100 000 person-years is zero |
| 3 | Noise | Proportion of the population ever exposed to noise greater than 85 decibels at work or through their occupation | Background noise exposure |
| 3 | Particulate matter, gases, and fumes | Proportion of the population ever exposed to particulates, gases, or fumes at work or through their occupation | No occupational exposure to particulates, gases, or fumes |

*All occupational risk exposures were estimated for ages 15 and older.

**Table S2.** List of International Classification of Diseases (ICD) codes mapped to the Global Burden of Disease cause list for causes of death

| **Cause** | **ICD10** | **ICD9** |
| --- | --- | --- |
| **Chronic respiratory diseases (CRD)** | | |
| Chronic obstructive pulmonary disease | J41-J44.9 | 491-492.9, 496-499 |
| Asthma | J45-J46.9 | 493-493.9 |
| Pneumoconiosis | J60-J63.8, J65-J65.0, J92.0 | 500-504.9 |
| **Age-related and other hearing loss** | - | - |
| **Low back pain** | M54.3, M54.4, M54.5 | 724 |
| **Cancer** | | |
| Nasopharynx cancer | C11-C11.9, D10.6 | 147-147.9, 210.7-210.9 |
| Larynx cancer | C32-C32.9, D02.0, D14.1, D38.0 | 161-161.9, 212.1,231.0,235.6 |
| Tracheal, bronchus, and lung cancer | C33-C34.9, D02.1-D02.3, D14.2-D14.3, D38.1 | 162-162.9, 212.2-212.3, 231.1-231.2, 235.7 |
| Ovarian cancer | C56-C56.9, D27-D27.9, D39.1 | 183-183.0, 220-220.9, 236.2 |
| Kidney cancer | C64-C65.9, D30.0-D30.1, D41.0-D41.1 | 189.0-189.1, 189.5-189.6, 223.0-223.1 |
| Mesothelioma | C45-C45.9 |  |
| Leukemia | C91- C95.9 | 204-208.9 |
| **Injuries** | | |
| Road injuries | V01-V04.9, V06-V80.9, V82-V82.9, V87.2-V87.3 |  |
| Other transport injuries | V00-V00.8, V05-V05.9, V81-V81.9, V83-V86.9, V88.2-V88.3, V90-  V98.8 | E800-E807,  E830-E838, E840-E849 |
| Falls | W00-W19.9 | E880-E886, E888 |
| Drowning | W65-W70.9, W73-W74.9 | E910 |
| Fire, heat, and hot substances | X00-X06.9, X08-X19.9 | E890-E899, E924 |
| Poisonings | X46-X48.9 | E856-E857, E861-E865, E867-E869 |
| Exposure to mechanical forces | W20-W38.9, W40-W43.9, W45.0-W45.2,  W46-W46.2, W49-W52 | E916-E922 |
| Animal contact | W52.0-W62.9, W64-W64.9, X20-X29.9 | E905-E906 |
| Foreign body | W44-W45, W45.3-W45.9, W75-W75.9, W78-  W80.9, W83-W84.9 | E911-E915 |
| Other unintentional injuries | W39-W39.9, W77-W77.9, W81-W81.9, W85-W87.9,  X50-X54.9, X57-X58.9 | E903-E904, E923, E925, E927-E928 |

**Table S3.** Epidemiological evidences supporting causality between occupational risk-outcome pairs included in the GBD 2017

| Occupational Risk | Outcome | Citation/Note |
| --- | --- | --- |
| Asbestos | Larynx cancer | Goodman M, Morgan RW, Ray R, Malloy CD, Zhao K. Cancer in asbestos-exposed occupational cohorts: a meta-analysis. Cancer Causes Control 1999; 10: 453–65. |
| Asbestos | Tracheal, bronchus and lung cancer | Lenters V, Vermeulen R, Dogger S, et al. A meta-analysis of asbestos and lung cancer: is better quality exposure assessment associated with steeper slopes of the exposure-response relationships? Environ Health Perspect 2011; 119: 1547–55. |
| Asbestos | Ovarian cancer | Camargo MC, Stayner LT, Straif K, et al. Occupational exposure to asbestos and ovarian cancer: a meta analysis. Environ Health Perspect 2011; 119: 1211–7. |
| Asbestos | Mesothelioma | Bourdès V, Boffetta P, Pisani P. Environmental exposure to asbestos and risk of pleural mesothelioma: review and meta-analysis. Eur J Epidemiol 2000; 16: 411–7. |
| Arsenic | Tracheal, bronchus and lung cancer | Lenters V, Vermeulen R, Dogger S, et al. A meta-analysis of asbestos and lung cancer: is better quality exposure assessment associated with steeper slopes of the exposure-response relationships? Environ Health Perspect 2011; 119: 1547–55. |
| Benzene | Leukemia | Khalade A, Jaakkola MS, Pukkala E, Jaakkola JJK. Exposure to benzene at work and the risk of leukemia: a systematic review and meta-analysis. Environ Health 2010; 9: 31 |
| Beryllium | Tracheal, bronchus and lung cancer | Boffetta P, Fryzek JP, Mandel JS. Occupational exposure to beryllium and cancer risk: a review of the epidemiologic evidence. Crit Rev Toxicol 2012; 42: 107–18. |
| Cadmium | Tracheal, bronchus and lung cancer | Verougstraete V, Lison D, Hotz P. Cadmium, lung and prostate cancer: a systematic review of recent epidemiological data. J Toxicol Environ Health B Crit Rev 2003; 6: 227–55. |
| Chromium | Tracheal, bronchus and lung cancer | Denis Ambroise, Pascal Wild and Jean-Jacques Moulin, Scandinavian Journal of Work, Environment & Health, Vol. 32, No. 1 (February 2006), pp. 22-31 |
| Diesel engine exhaust | Tracheal, bronchus and lung cancer | Lipsett M, Campleman S. Occupational exposure to diesel exhaust and lung cancer: a meta-analysis. Am J Public Health 1999; 89: 1009–17. |
| Formaldehyde | Nasopharynx cancer | Hauptmann M, Lubin JH, Stewart PA, Hayes RB, Blair A. Mortality from solid cancers among workers in formaldehyde industries. Am J Epidemiol 2004; 159: 1117–30. |
| Formaldehyde | Leukemia | Collins JJ, Lineker GA. A review and meta-analysis of formaldehyde exposure and leukemia. Regul Toxicol Pharmacol 2004; 40: 81–91. |
| Nickel | Tracheal, bronchus and lung cancer | Grimsrud TK, Berge SR, Haldorsen T, Andersen A. Can lung cancer risk among nickel refinery workers be explained by occupational exposures other than nickel? Epidemiology 2005; 16: 146–54. |
| Polycyclic aromatic  hydrocarbons | Tracheal, bronchus and lung cancer | Armstrong B, Hutchinson E, Unwin J, Fletcher T. Lung cancer risk after exposure to polycyclic aromatic hydrocarbons: a review and meta-analysis. Environ Health Perspect 2004; 112: 970–8. |
| Silica | Tracheal, bronchus and lung cancer | Liu Y, Steenland K, Rong Y, Hnizdo E, Huang X, Zhang H, et al. Exposure-Response Analysis and Risk Assessment for Lung Cancer in Relationship to Silica Exposure: A 44-Year Cohort Study of 34,018 Workers. Am J Epidemiol. 2013 Nov 1;178(9):1424–33. |
| Sulfuric acid | Larynx cancer | Soskolne CL, Jhangri GS, Siemiatycki J, et al. Occupational exposure to sulfuric acid in southern Ontario, Canada, in association with laryngeal cancer. Scand J Work Environ Health 1992; 18: 225–32. |
| Trichloroethylene | Kidney cancer | Kelsh MA, Alexander DD, Mink PJ, Mandel JH. Occupational trichloroethylene exposure and kidney cancer: a meta-analysis. Epidemiology 2010; 21: 95–102. |
| Asthmagens | Asthma | Karjalainen A, Kurppa K, Martikainen R, Klaukka T, Karjalainen J. Work is related to a substantial portion of adult-onset asthma incidence in the Finnish population. Am J Respir Crit Care Med 2001; 164: 565–8. |
| Particulate matter, gases, and fumes | Chronic obstructive pulmonary  disease | Blanc PD, Iribarren C, Trupin L, et al. Occupational exposures and the risk of COPD: dusty trades revisited. Thorax 2009; 64: 6–12. |
| Noise | Age-related and other hearing loss | Agrawal Y, Platz EA, Niparko JK. Prevalence of hearing loss and differences by demographic characteristics among US adults: data from the National Health and Nutrition Examination Survey, 1999-2004. Arch Intern Med 2008; 168: 1522–30. |
| Noise | Age-related and other hearing loss | Davis A. The prevalence of hearing impairment and reported hearing disability among adults in Great Britain. International Journal of Epidemiology 1989,18: 911-917. |
| Noise | Age-related and other hearing loss | Wilson D, Walsh P, Sanchez L, Davis A, Taylor A, Tucker G, Meagher I. The epidemiology of hearing impairment in an Australian adult population. International Journal of Epidemiology 1999; 28:247-252. |
| Injuries | Injuries | International Labour Organization. Resolution concerning statistics of occupational injuries (resulting from occupational accidents). 1998; published online Oct. http://www.ilo.org/global/statistics-anddatabases/standards-and-guidelines/resolutions-adopted-by-international-conferences-of-labourstatisticians/ WCMS_087528/lang--en/index.htm. |
| Injuries | Injuries | Eurostat. Accidents at work statistics. http://ec.europa.eu/eurostat/statisticsexplained/index.php/Accidents_at_work_statistics. |
| Ergonomic factors | Low back pain | Driscoll T, Jacklyn G, Orchard J, et al. The global burden of occupationally related low back pain: estimates from the Global Burden of Disease 2010 study. Ann Rheum Dis 2014; 73: 975–81. |

**Table S4.** Relative risks used by age and sex for each outcome for occupational exposure to carcinogens, asthmagens and particulate matter, gases, and fumes

| **Risk - Outcome** | **Category** | **Morbidity / Mortality** | **Sex** | **Age group** | | | | | | | | | | | | | | | | |
| --- | --- | --- | --- | --- | --- | --- | --- | --- | --- | --- | --- | --- | --- | --- | --- | --- | --- | --- | --- | --- |
|  |  |  |  | **15-19**  **years** | **20-24**  **years** | **25-29**  **years** | **30-34**  **years** | **35-39**  **years** | **40-44**  **years** | **45-49**  **years** | **50-54**  **years** | **55-59**  **years** | **60-64**  **years** | **65-69**  **years** | **70-74**  **years** | **75-79**  **years** | **80-84**  **years** | **85-89**  **years** | **90-94**  **years** | **95+**  **years** |
| **Occupational exposure to asbestos** | | | | | | | | | | | | | | | | | | | | |
| Larynx cancer | High exposure | Both | Males | 1.38 (1.18, 1.612) | 1.38 (1.18, 1.612) | 1.38 (1.18, 1.612) | 1.38 (1.18, 1.612) | 1.38 (1.18, 1.612) | 1.38 (1.18, 1.612) | 1.38 (1.18, 1.612) | 1.38 (1.18, 1.612) | 1.38 (1.18, 1.612) | 1.38 (1.18, 1.612) | 1.38 (1.18, 1.612) | 1.38 (1.18, 1.612) | 1.38 (1.18, 1.612) | 1.38 (1.18, 1.612) | 1.38 (1.18, 1.612) | 1.38 (1.18, 1.612) | 1.38 (1.18, 1.612) |
|  | High exposure | Both | Females | 1.385 (1.18, 1.598) | 1.385 (1.18, 1.598) | 1.385 (1.18, 1.598) | 1.385 (1.18, 1.598) | 1.385 (1.18, 1.598) | 1.385 (1.18, 1.598) | 1.385 (1.18, 1.598) | 1.385 (1.18, 1.598) | 1.385 (1.18, 1.598) | 1.385 (1.18, 1.598) | 1.385 (1.18, 1.598) | 1.385 (1.18, 1.598) | 1.385 (1.18, 1.598) | 1.385 (1.18, 1.598) | 1.385 (1.18, 1.598) | 1.385 (1.18, 1.598) | 1.385 (1.18, 1.598) |
|  | Low exposure | Both | Males | 1.0 (1.0, 1.0) | 1.0 (1.0, 1.0) | 1.0 (1.0, 1.0) | 1.0 (1.0, 1.0) | 1.0 (1.0, 1.0) | 1.0 (1.0, 1.0) | 1.0 (1.0, 1.0) | 1.0 (1.0, 1.0) | 1.0 (1.0, 1.0) | 1.0 (1.0, 1.0) | 1.0 (1.0, 1.0) | 1.0 (1.0, 1.0) | 1.0 (1.0, 1.0) | 1.0 (1.0, 1.0) | 1.0 (1.0, 1.0) | 1.0 (1.0, 1.0) | 1.0 (1.0, 1.0) |
|  | Low exposure | Both | Females | 1.0 (1.0, 1.0) | 1.0 (1.0, 1.0) | 1.0 (1.0, 1.0) | 1.0 (1.0, 1.0) | 1.0 (1.0, 1.0) | 1.0 (1.0, 1.0) | 1.0 (1.0, 1.0) | 1.0 (1.0, 1.0) | 1.0 (1.0, 1.0) | 1.0 (1.0, 1.0) | 1.0 (1.0, 1.0) | 1.0 (1.0, 1.0) | 1.0 (1.0, 1.0) | 1.0 (1.0, 1.0) | 1.0 (1.0, 1.0) | 1.0 (1.0, 1.0) | 1.0 (1.0, 1.0) |
| Tracheal, bronchus, and lung cancer | High exposure | Both | Males | 2.279 (1.74, 2.936) | 2.279 (1.74, 2.936) | 2.279 (1.74, 2.936) | 2.279 (1.74, 2.936) | 2.279 (1.74, 2.936) | 2.279 (1.74, 2.936) | 2.279 (1.74, 2.936) | 2.279 (1.74, 2.936) | 2.279 (1.74, 2.936) | 2.279 (1.74, 2.936) | 2.279 (1.74, 2.936) | 2.279 (1.74, 2.936) | 2.279 (1.74, 2.936) | 2.279 (1.74, 2.936) | 2.279 (1.74, 2.936) | 2.279 (1.74, 2.936) | 2.279 (1.74, 2.936) |
|  | High exposure | Both | Females | 1.875 (1.589, 2.208) | 1.875 (1.589, 2.208) | 1.875 (1.589, 2.208) | 1.875 (1.589, 2.208) | 1.875 (1.589, 2.208) | 1.875 (1.589, 2.208) | 1.875 (1.589, 2.208) | 1.875 (1.589, 2.208) | 1.875 (1.589, 2.208) | 1.875 (1.589, 2.208) | 1.875 (1.589, 2.208) | 1.875 (1.589, 2.208) | 1.875 (1.589, 2.208) | 1.875 (1.589, 2.208) | 1.875 (1.589, 2.208) | 1.875 (1.589, 2.208) | 1.875 (1.58, 2.208) |
|  | Low exposure | Both | Males | 1.0 (1.0, 1.0) | 1.0 (1.0, 1.0) | 1.0 (1.0, 1.0) | 1.0 (1.0, 1.0) | 1.0 (1.0, 1.0) | 1.0 (1.0, 1.0) | 1.0 (1.0, 1.0) | 1.0 (1.0, 1.0) | 1.0 (1.0, 1.0) | 1.0 (1.0, 1.0) | 1.0 (1.0, 1.0) | 1.0 (1.0, 1.0) | 1.0 (1.0, 1.0) | 1.0 (1.0, 1.0) | 1.0 (1.0, 1.0) | 1.0 (1.0, 1.0) | 1.0 (1.0, 1.0) |
|  | Low exposure | Both | Females | 1.0 (1.0, 1.0) | 1.0 (1.0, 1.0) | 1.0 (1.0, 1.0) | 1.0 (1.0, 1.0) | 1.0 (1.0, 1.0) | 1.0 (1.0, 1.0) | 1.0 (1.0, 1.0) | 1.0 (1.0, 1.0) | 1.0 (1.0, 1.0) | 1.0 (1.0, 1.0) | 1.0 (1.0, 1.0) | 1.0 (1.0, 1.0) | 1.0 (1.0, 1.0) | 1.0 (1.0, 1.0) | 1.0 (1.0, 1.0) | 1.0 (1.0, 1.0) | 1.0 (1.0, 1.0) |
| Ovarian cancer | High exposure | Both | Both | 1.811 (1.385, 2.306) | 1.811 (1.385, 2.306) | 1.811 (1.385, 2.306) | 1.811 (1.385, 2.306) | 1.811 (1.385, 2.306) | 1.811 (1.385, 2.306) | 1.811 (1.385, 2.306) | 1.811 (1.385, 2.306) | 1.811 (1.385, 2.306) | 1.811 (1.385, 2.306) | 1.811 (1.385, 2.306) | 1.811 (1.385, 2.306) | 1.811 (1.385, 2.306) | 1.811 (1.385, 2.306) | 1.811 (1.385, 2.306) | 1.811 (1.385, 2.306) | 1.811 (1.38, 2.306) |
|  | Low exposure | Both | Both | 1.0 (1.0, 1.0) | 1.0 (1.0, 1.0) | 1.0 (1.0, 1.0) | 1.0 (1.0, 1.0) | 1.0 (1.0, 1.0) | 1.0 (1.0, 1.0) | 1.0 (1.0, 1.0) | 1.0 (1.0, 1.0) | 1.0 (1.0, 1.0) | 1.0 (1.0, 1.0) | 1.0 (1.0, 1.0) | 1.0 (1.0, 1.0) | 1.0 (1.0, 1.0) | 1.0 (1.0, 1.0) | 1.0 (1.0, 1.0) | 1.0 (1.0, 1.0) | 1.0 (1.0, 1.0) |
| **Occupational exposure to arsenic** | | | | | | | | | | | | | | | | | | | | |
| Tracheal, bronchus, and lung cancer | High exposure | Both | Both | 2.061 (1.521, 2.553) | 2.061 (1.521, 2.553) | 2.061 (1.521, 2.553) | 2.061 (1.521, 2.553) | 2.061 (1.521, 2.553) | 2.061 (1.521, 2.553) | 2.061 (1.521, 2.553) | 2.061 (1.521, 2.553) | 2.061 (1.521, 2.553) | 2.061 (1.521, 2.553) | 2.061 (1.521, 2.553) | 2.061 (1.521, 2.553) | 2.061 (1.521, 2.553) | 2.061 (1.521, 2.553) | 2.061 (1.521, 2.553) | 2.061 (1.521, 2.553) | 2.061 (1.52, 2.553) |
|  | Low exposure | Both | Both | 1.749 (0.698, 2.775) | 1.749 (0.698, 2.775) | 1.749 (0.698, 2.775) | 1.749 (0.698, 2.775) | 1.749 (0.698, 2.775) | 1.749 (0.698, 2.775) | 1.749 (0.698, 2.775) | 1.749 (0.698, 2.775) | 1.749 (0.698, 2.775) | 1.749 (0.698, 2.775) | 1.749 (0.698, 2.775) | 1.749 (0.698, 2.775) | 1.749 (0.698, 2.775) | 1.749 (0.698, 2.775) | 1.749 (0.698, 2.775) | 1.749 (0.698, 2.775) | 1.749 (0.69, 2.775) |
|  | No exposure | Both | Both | 1.0 (1.0, 1.0) | 1.0 (1.0, 1.0) | 1.0 (1.0, 1.0) | 1.0 (1.0, 1.0) | 1.0 (1.0, 1.0) | 1.0 (1.0, 1.0) | 1.0 (1.0, 1.0) | 1.0 (1.0, 1.0) | 1.0 (1.0, 1.0) | 1.0 (1.0, 1.0) | 1.0 (1.0, 1.0) | 1.0 (1.0, 1.0) | 1.0 (1.0, 1.0) | 1.0 (1.0, 1.0) | 1.0 (1.0, 1.0) | 1.0 (1.0, 1.0) | 1.0 (1.0, 1.0) |
| **Occupational exposure to benzene** | | | | | | | | | | | | | | | | | | | | |
| Acute lymphoid leukemia | High exposure | Both | Both | 2.623 (1.222, 3.975) | 2.623 (1.222, 3.975) | 2.623 (1.222, 3.975) | 2.623 (1.222, 3.975) | 2.623 (1.222, 3.975) | 2.623 (1.222, 3.975) | 2.623 (1.222, 3.975) | 2.623 (1.222, 3.975) | 2.623 (1.222, 3.975) | 2.623 (1.222, 3.975) | 2.623 (1.222, 3.975) | 2.623 (1.222, 3.975) | 2.623 (1.222, 3.975) | 2.623 (1.222, 3.975) | 2.623 (1.222, 3.975) | 2.623 (1.222, 3.975) | 2.623 (1.22, 3.975) |
|  | Low exposure | Both | Both | 1.626 (0.998, 2.256) | 1.626 (0.998, 2.256) | 1.626 (0.998, 2.256) | 1.626 (0.998, 2.256) | 1.626 (0.998, 2.256) | 1.626 (0.998, 2.256) | 1.626 (0.998, 2.256) | 1.626 (0.998, 2.256) | 1.626 (0.998, 2.256) | 1.626 (0.998, 2.256) | 1.626 (0.998, 2.256) | 1.626 (0.998, 2.256) | 1.626 (0.998, 2.256) | 1.626 (0.998, 2.256) | 1.626 (0.998, 2.256) | 1.626 (0.998, 2.256) | 1.626 (0.99, 2.256) |
|  | No exposure | Both | Both | 1.0 (1.0, 1.0) | 1.0 (1.0, 1.0) | 1.0 (1.0, 1.0) | 1.0 (1.0, 1.0) | 1.0 (1.0, 1.0) | 1.0 (1.0, 1.0) | 1.0 (1.0, 1.0) | 1.0 (1.0, 1.0) | 1.0 (1.0, 1.0) | 1.0 (1.0, 1.0) | 1.0 (1.0, 1.0) | 1.0 (1.0, 1.0) | 1.0 (1.0, 1.0) | 1.0 (1.0, 1.0) | 1.0 (1.0, 1.0) | 1.0 (1.0, 1.0) | 1.0 (1.0, 1.0) |
| Chronic lymphoid leukemia | High exposure | Both | Both | 2.623 (1.222, 3.975) | 2.623 (1.222, 3.975) | 2.623 (1.222, 3.975) | 2.623 (1.222, 3.975) | 2.623 (1.222, 3.975) | 2.623 (1.222, 3.975) | 2.623 (1.222, 3.975) | 2.623 (1.222, 3.975) | 2.623 (1.222, 3.975) | 2.623 (1.222, 3.975) | 2.623 (1.222, 3.975) | 2.623 (1.222, 3.975) | 2.623 (1.222, 3.975) | 2.623 (1.222, 3.975) | 2.623 (1.222, 3.975) | 2.623 (1.222, 3.975) | 2.623 (1.22, 3.975) |
|  | Low exposure | Both | Both | 1.626 (0.998, 2.256) | 1.626 (0.998, 2.256) | 1.626 (0.998, 2.256) | 1.626 (0.998, 2.256) | 1.626 (0.998, 2.256) | 1.626 (0.998, 2.256) | 1.626 (0.998, 2.256) | 1.626 (0.998, 2.256) | 1.626 (0.998, 2.256) | 1.626 (0.998, 2.256) | 1.626 (0.998, 2.256) | 1.626 (0.998, 2.256) | 1.626 (0.998, 2.256) | 1.626 (0.998, 2.256) | 1.626 (0.998, 2.256) | 1.626 (0.998, 2.256) | 1.626 (0.99, 2.256) |
|  | No exposure | Both | Both | 1.0 (1.0, 1.0) | 1.0 (1.0, 1.0) | 1.0 (1.0, 1.0) | 1.0 (1.0, 1.0) | 1.0 (1.0, 1.0) | 1.0 (1.0, 1.0) | 1.0 (1.0, 1.0) | 1.0 (1.0, 1.0) | 1.0 (1.0, 1.0) | 1.0 (1.0, 1.0) | 1.0 (1.0, 1.0) | 1.0 (1.0, 1.0) | 1.0 (1.0, 1.0) | 1.0 (1.0, 1.0) | 1.0 (1.0, 1.0) | 1.0 (1.0, 1.0) | 1.0 (1.0, 1.0) |
| Acute myeloid leukemia | High exposure | Both | Both | 2.623 (1.222, 3.975) | 2.623 (1.222, 3.975) | 2.623 (1.222, 3.975) | 2.623 (1.222, 3.975) | 2.623 (1.222, 3.975) | 2.623 (1.222, 3.975) | 2.623 (1.222, 3.975) | 2.623 (1.222, 3.975) | 2.623 (1.222, 3.975) | 2.623 (1.222, 3.975) | 2.623 (1.222, 3.975) | 2.623 (1.222, 3.975) | 2.623 (1.222, 3.975) | 2.623 (1.222, 3.975) | 2.623 (1.222, 3.975) | 2.623 (1.222, 3.975) | 2.623 (1.22, 3.975) |
|  | Low exposure | Both | Both | 1.626 (0.998, 2.256) | 1.626 (0.998, 2.256) | 1.626 (0.998, 2.256) | 1.626 (0.998, 2.256) | 1.626 (0.998, 2.256) | 1.626 (0.998, 2.256) | 1.626 (0.998, 2.256) | 1.626 (0.998, 2.256) | 1.626 (0.998, 2.256) | 1.626 (0.998, 2.256) | 1.626 (0.998, 2.256) | 1.626 (0.998, 2.256) | 1.626 (0.998, 2.256) | 1.626 (0.998, 2.256) | 1.626 (0.998, 2.256) | 1.626 (0.998, 2.256) | 1.626 (0.99, 2.256) |
|  | No exposure | Both | Both | 1.0 (1.0, 1.0) | 1.0 (1.0, 1.0) | 1.0 (1.0, 1.0) | 1.0 (1.0, 1.0) | 1.0 (1.0, 1.0) | 1.0 (1.0, 1.0) | 1.0 (1.0, 1.0) | 1.0 (1.0, 1.0) | 1.0 (1.0, 1.0) | 1.0 (1.0, 1.0) | 1.0 (1.0, 1.0) | 1.0 (1.0, 1.0) | 1.0 (1.0, 1.0) | 1.0 (1.0, 1.0) | 1.0 (1.0, 1.0) | 1.0 (1.0, 1.0) | 1.0 (1.0, 1.0) |
| Chronic myeloid leukemia | High exposure | Both | Both | 2.623 (1.222, 3.975) | 2.623 (1.222, 3.975) | 2.623 (1.222, 3.975) | 2.623 (1.222, 3.975) | 2.623 (1.222, 3.975) | 2.623 (1.222, 3.975) | 2.623 (1.222, 3.975) | 2.623 (1.222, 3.975) | 2.623 (1.222, 3.975) | 2.623 (1.222, 3.975) | 2.623 (1.222, 3.975) | 2.623 (1.222, 3.975) | 2.623 (1.222, 3.975) | 2.623 (1.222, 3.975) | 2.623 (1.222, 3.975) | 2.623 (1.222, 3.975) | 2.623 (1.22, 3.975) |
|  | Low exposure | Both | Both | 1.626 (0.998, 2.256) | 1.626 (0.998, 2.256) | 1.626 (0.998, 2.256) | 1.626 (0.998, 2.256) | 1.626 (0.998, 2.256) | 1.626 (0.998, 2.256) | 1.626 (0.998, 2.256) | 1.626 (0.998, 2.256) | 1.626 (0.998, 2.256) | 1.626 (0.998, 2.256) | 1.626 (0.998, 2.256) | 1.626 (0.998, 2.256) | 1.626 (0.998, 2.256) | 1.626 (0.998, 2.256) | 1.626 (0.998, 2.256) | 1.626 (0.998, 2.256) | 1.626 (0.99, 2.256) |
|  | No exposure | Both | Both | 1.0 (1.0, 1.0) | 1.0 (1.0, 1.0) | 1.0 (1.0, 1.0) | 1.0 (1.0, 1.0) | 1.0 (1.0, 1.0) | 1.0 (1.0, 1.0) | 1.0 (1.0, 1.0) | 1.0 (1.0, 1.0) | 1.0 (1.0, 1.0) | 1.0 (1.0, 1.0) | 1.0 (1.0, 1.0) | 1.0 (1.0, 1.0) | 1.0 (1.0, 1.0) | 1.0 (1.0, 1.0) | 1.0 (1.0, 1.0) | 1.0 (1.0, 1.0) | 1.0 (1.0, 1.0) |
| Other leukemia | High exposure | Both | Both | 2.623 (1.222, 3.975) | 2.623 (1.222, 3.975) | 2.623 (1.222, 3.975) | 2.623 (1.222, 3.975) | 2.623 (1.222, 3.975) | 2.623 (1.222, 3.975) | 2.623 (1.222, 3.975) | 2.623 (1.222, 3.975) | 2.623 (1.222, 3.975) | 2.623 (1.222, 3.975) | 2.623 (1.222, 3.975) | 2.623 (1.222, 3.975) | 2.623 (1.222, 3.975) | 2.623 (1.222, 3.975) | 2.623 (1.222, 3.975) | 2.623 (1.222, 3.975) | 2.623 (1.22, 3.975) |
|  | Low exposure | Both | Both | 1.626 (0.998, 2.256) | 1.626 (0.998, 2.256) | 1.626 (0.998, 2.256) | 1.626 (0.998, 2.256) | 1.626 (0.998, 2.256) | 1.626 (0.998, 2.256) | 1.626 (0.998, 2.256) | 1.626 (0.998, 2.256) | 1.626 (0.998, 2.256) | 1.626 (0.998, 2.256) | 1.626 (0.998, 2.256) | 1.626 (0.998, 2.256) | 1.626 (0.998, 2.256) | 1.626 (0.998, 2.256) | 1.626 (0.998, 2.256) | 1.626 (0.998, 2.256) | 1.626 (0.99, 2.256) |
|  | No exposure | Both | Both | 1.0 (1.0, 1.0) | 1.0 (1.0, 1.0) | 1.0 (1.0, 1.0) | 1.0 (1.0, 1.0) | 1.0 (1.0, 1.0) | 1.0 (1.0, 1.0) | 1.0 (1.0, 1.0) | 1.0 (1.0, 1.0) | 1.0 (1.0, 1.0) | 1.0 (1.0, 1.0) | 1.0 (1.0, 1.0) | 1.0 (1.0, 1.0) | 1.0 (1.0, 1.0) | 1.0 (1.0, 1.0) | 1.0 (1.0, 1.0) | 1.0 (1.0, 1.0) | 1.0 (1.0, 1.0) |
| **Occupational exposure to beryllium** | | | | | | | | | | | | | | | | | | | | |
| Tracheal, bronchus, and lung cancer | High exposure | Both | Males | 1.174 (1.086, 1.269) | 1.169 (1.065, 1.276) | 1.17 (1.073, 1.274) | 1.169 (1.073, 1.269) | 1.172 (1.081, 1.271) | 1.168 (1.07, 1.276) | 1.171 (1.071, 1.274) | 1.171 (1.075, 1.273) | 1.17 (1.073, 1.271) | 1.171 (1.072, 1.271) | 1.172 (1.081, 1.276) | 1.171 (1.076, 1.27) | 1.174 (1.079, 1.276) | 1.171 (1.072, 1.273) | 1.171 (1.072, 1.273) | 1.171 (1.072, 1.273) | 1.171 (1.07, 1.273) |
|  | High exposure | Both | Females | 1.17 (1.082, 1.262) | 1.17 (1.076, 1.277) | 1.169 (1.072, 1.277) | 1.17 (1.077, 1.275) | 1.172 (1.075, 1.274) | 1.171 (1.078, 1.276) | 1.173 (1.074, 1.276) | 1.172 (1.074, 1.273) | 1.173 (1.081, 1.277) | 1.169 (1.074, 1.269) | 1.173 (1.077, 1.282) | 1.167 (1.069, 1.269) | 1.169 (1.073, 1.274) | 1.171 (1.068, 1.279) | 1.171 (1.068, 1.279) | 1.171 (1.068, 1.279) | 1.171 (1.06, 1.279) |
|  | Low exposure | Both | Males | 1.0 (1.0, 1.0) | 1.0 (1.0, 1.0) | 1.0 (1.0, 1.0) | 1.0 (1.0, 1.0) | 1.0 (1.0, 1.0) | 1.0 (1.0, 1.0) | 1.0 (1.0, 1.0) | 1.0 (1.0, 1.0) | 1.0 (1.0, 1.0) | 1.0 (1.0, 1.0) | 1.0 (1.0, 1.0) | 1.0 (1.0, 1.0) | 1.0 (1.0, 1.0) | 1.0 (1.0, 1.0) | 1.0 (1.0, 1.0) | 1.0 (1.0, 1.0) | 1.0 (1.0, 1.0) |
|  | Low exposure | Both | Females | 1.0 (1.0, 1.0) | 1.0 (1.0, 1.0) | 1.0 (1.0, 1.0) | 1.0 (1.0, 1.0) | 1.0 (1.0, 1.0) | 1.0 (1.0, 1.0) | 1.0 (1.0, 1.0) | 1.0 (1.0, 1.0) | 1.0 (1.0, 1.0) | 1.0 (1.0, 1.0) | 1.0 (1.0, 1.0) | 1.0 (1.0, 1.0) | 1.0 (1.0, 1.0) | 1.0 (1.0, 1.0) | 1.0 (1.0, 1.0) | 1.0 (1.0, 1.0) | 1.0 (1.0, 1.0) |
|  | No exposure | Both | Males | 1.0 (1.0, 1.0) | 1.0 (1.0, 1.0) | 1.0 (1.0, 1.0) | 1.0 (1.0, 1.0) | 1.0 (1.0, 1.0) | 1.0 (1.0, 1.0) | 1.0 (1.0, 1.0) | 1.0 (1.0, 1.0) | 1.0 (1.0, 1.0) | 1.0 (1.0, 1.0) | 1.0 (1.0, 1.0) | 1.0 (1.0, 1.0) | 1.0 (1.0, 1.0) | 1.0 (1.0, 1.0) | 1.0 (1.0, 1.0) | 1.0 (1.0, 1.0) | 1.0 (1.0, 1.0) |
|  | No exposure | Both | Females | 1.0 (1.0, 1.0) | 1.0 (1.0, 1.0) | 1.0 (1.0, 1.0) | 1.0 (1.0, 1.0) | 1.0 (1.0, 1.0) | 1.0 (1.0, 1.0) | 1.0 (1.0, 1.0) | 1.0 (1.0, 1.0) | 1.0 (1.0, 1.0) | 1.0 (1.0, 1.0) | 1.0 (1.0, 1.0) | 1.0 (1.0, 1.0) | 1.0 (1.0, 1.0) | 1.0 (1.0, 1.0) | 1.0 (1.0, 1.0) | 1.0 (1.0, 1.0) | 1.0 (1.0, 1.0) |
| **Occupational exposure to cadmium** | | | | | | | | | | | | | | | | | | | | |
| Tracheal, bronchus, and lung cancer | High exposure | Both | Males | 1.192 (1.097, 1.292) | 1.188 (1.083, 1.287) | 1.19 (1.102, 1.295) | 1.191 (1.092, 1.304) | 1.19 (1.091, 1.298) | 1.19 (1.092, 1.297) | 1.192 (1.096, 1.298) | 1.193 (1.096, 1.296) | 1.19 (1.089, 1.293) | 1.192 (1.089, 1.297) | 1.193 (1.088, 1.3) | 1.187 (1.095, 1.29) | 1.191 (1.095, 1.289) | 1.19 (1.088, 1.296) | 1.19 (1.088, 1.296) | 1.19 (1.088, 1.296) | 1.19 (1.08, 1.296) |
|  | High exposure | Both | Females | 1.191 (1.087, 1.295) | 1.191 (1.099, 1.29) | 1.188 (1.093, 1.293) | 1.19 (1.092, 1.302) | 1.191 (1.089, 1.292) | 1.194 (1.098, 1.297) | 1.188 (1.091, 1.3) | 1.19 (1.095, 1.291) | 1.19 (1.096, 1.294) | 1.189 (1.095, 1.29) | 1.192 (1.095, 1.296) | 1.192 (1.09, 1.298) | 1.193 (1.097, 1.297) | 1.193 (1.1, 1.301) | 1.193 (1.1, 1.301) | 1.193 (1.1, 1.301) | 1.193 (1.1, 1.301) |
|  | Low exposure | Both | Males | 1.0 (1.0, 1.0) | 1.0 (1.0, 1.0) | 1.0 (1.0, 1.0) | 1.0 (1.0, 1.0) | 1.0 (1.0, 1.0) | 1.0 (1.0, 1.0) | 1.0 (1.0, 1.0) | 1.0 (1.0, 1.0) | 1.0 (1.0, 1.0) | 1.0 (1.0, 1.0) | 1.0 (1.0, 1.0) | 1.0 (1.0, 1.0) | 1.0 (1.0, 1.0) | 1.0 (1.0, 1.0) | 1.0 (1.0, 1.0) | 1.0 (1.0, 1.0) | 1.0 (1.0, 1.0) |
|  | Low exposure | Both | Females | 1.0 (1.0, 1.0) | 1.0 (1.0, 1.0) | 1.0 (1.0, 1.0) | 1.0 (1.0, 1.0) | 1.0 (1.0, 1.0) | 1.0 (1.0, 1.0) | 1.0 (1.0, 1.0) | 1.0 (1.0, 1.0) | 1.0 (1.0, 1.0) | 1.0 (1.0, 1.0) | 1.0 (1.0, 1.0) | 1.0 (1.0, 1.0) | 1.0 (1.0, 1.0) | 1.0 (1.0, 1.0) | 1.0 (1.0, 1.0) | 1.0 (1.0, 1.0) | 1.0 (1.0, 1.0) |
|  | No exposure | Both | Males | 1.0 (1.0, 1.0) | 1.0 (1.0, 1.0) | 1.0 (1.0, 1.0) | 1.0 (1.0, 1.0) | 1.0 (1.0, 1.0) | 1.0 (1.0, 1.0) | 1.0 (1.0, 1.0) | 1.0 (1.0, 1.0) | 1.0 (1.0, 1.0) | 1.0 (1.0, 1.0) | 1.0 (1.0, 1.0) | 1.0 (1.0, 1.0) | 1.0 (1.0, 1.0) | 1.0 (1.0, 1.0) | 1.0 (1.0, 1.0) | 1.0 (1.0, 1.0) | 1.0 (1.0, 1.0) |
|  | No exposure | Both | Females | 1.0 (1.0, 1.0) | 1.0 (1.0, 1.0) | 1.0 (1.0, 1.0) | 1.0 (1.0, 1.0) | 1.0 (1.0, 1.0) | 1.0 (1.0, 1.0) | 1.0 (1.0, 1.0) | 1.0 (1.0, 1.0) | 1.0 (1.0, 1.0) | 1.0 (1.0, 1.0) | 1.0 (1.0, 1.0) | 1.0 (1.0, 1.0) | 1.0 (1.0, 1.0) | 1.0 (1.0, 1.0) | 1.0 (1.0, 1.0) | 1.0 (1.0, 1.0) | 1.0 (1.0, 1.0) |
| **Occupational exposure to chromium** | | | | | | | | | | | | | | | | | | | | |
| Tracheal, bronchus, and lung cancer | High exposure | Both | Males | 1.179 (1.114, 1.245) | 1.181 (1.117, 1.25) | 1.181 (1.118, 1.249) | 1.18 (1.117, 1.244) | 1.183 (1.117, 1.249) | 1.18 (1.117, 1.239) | 1.18 (1.117, 1.249) | 1.182 (1.12, 1.246) | 1.181 (1.119, 1.246) | 1.179 (1.115, 1.242) | 1.182 (1.121, 1.247) | 1.18 (1.118, 1.248) | 1.18 (1.113, 1.245) | 1.181 (1.117, 1.244) | 1.181 (1.117, 1.244) | 1.181 (1.117, 1.244) | 1.181 (1.11, 1.244) |
|  | High exposure | Both | Females | 1.179 (1.116, 1.247) | 1.18 (1.115, 1.248) | 1.18 (1.115, 1.244) | 1.179 (1.118, 1.243) | 1.18 (1.116, 1.248) | 1.181 (1.115, 1.245) | 1.181 (1.119, 1.25) | 1.181 (1.118, 1.245) | 1.181 (1.117, 1.248) | 1.181 (1.117, 1.244) | 1.179 (1.121, 1.245) | 1.181 (1.116, 1.254) | 1.181 (1.116, 1.245) | 1.179 (1.113, 1.247) | 1.179 (1.113, 1.247) | 1.179 (1.113, 1.247) | 1.179 (1.11, 1.247) |
|  | Low exposure | Both | Males | 1.0 (1.0, 1.0) | 1.0 (1.0, 1.0) | 1.0 (1.0, 1.0) | 1.0 (1.0, 1.0) | 1.0 (1.0, 1.0) | 1.0 (1.0, 1.0) | 1.0 (1.0, 1.0) | 1.0 (1.0, 1.0) | 1.0 (1.0, 1.0) | 1.0 (1.0, 1.0) | 1.0 (1.0, 1.0) | 1.0 (1.0, 1.0) | 1.0 (1.0, 1.0) | 1.0 (1.0, 1.0) | 1.0 (1.0, 1.0) | 1.0 (1.0, 1.0) | 1.0 (1.0, 1.0) |
|  | Low exposure | Both | Females | 1.0 (1.0, 1.0) | 1.0 (1.0, 1.0) | 1.0 (1.0, 1.0) | 1.0 (1.0, 1.0) | 1.0 (1.0, 1.0) | 1.0 (1.0, 1.0) | 1.0 (1.0, 1.0) | 1.0 (1.0, 1.0) | 1.0 (1.0, 1.0) | 1.0 (1.0, 1.0) | 1.0 (1.0, 1.0) | 1.0 (1.0, 1.0) | 1.0 (1.0, 1.0) | 1.0 (1.0, 1.0) | 1.0 (1.0, 1.0) | 1.0 (1.0, 1.0) | 1.0 (1.0, 1.0) |
|  | No exposure | Both | Males | 1.0 (1.0, 1.0) | 1.0 (1.0, 1.0) | 1.0 (1.0, 1.0) | 1.0 (1.0, 1.0) | 1.0 (1.0, 1.0) | 1.0 (1.0, 1.0) | 1.0 (1.0, 1.0) | 1.0 (1.0, 1.0) | 1.0 (1.0, 1.0) | 1.0 (1.0, 1.0) | 1.0 (1.0, 1.0) | 1.0 (1.0, 1.0) | 1.0 (1.0, 1.0) | 1.0 (1.0, 1.0) | 1.0 (1.0, 1.0) | 1.0 (1.0, 1.0) | 1.0 (1.0, 1.0) |
|  | No exposure | Both | Females | 1.0 (1.0, 1.0) | 1.0 (1.0, 1.0) | 1.0 (1.0, 1.0) | 1.0 (1.0, 1.0) | 1.0 (1.0, 1.0) | 1.0 (1.0, 1.0) | 1.0 (1.0, 1.0) | 1.0 (1.0, 1.0) | 1.0 (1.0, 1.0) | 1.0 (1.0, 1.0) | 1.0 (1.0, 1.0) | 1.0 (1.0, 1.0) | 1.0 (1.0, 1.0) | 1.0 (1.0, 1.0) | 1.0 (1.0, 1.0) | 1.0 (1.0, 1.0) | 1.0 (1.0, 1.0) |
| **Occupational exposure to diesel engine exhaust** | | | | | | | | | | | | | | | | | | | | |
| Tracheal, bronchus, and lung cancer | High exposure | Both | Males | 1.469 (1.294, 1.658) | 1.477 (1.301, 1.665) | 1.473 (1.29, 1.668) | 1.474 (1.293, 1.675) | 1.472 (1.301, 1.669) | 1.47 (1.294, 1.662) | 1.475 (1.3, 1.667) | 1.469 (1.301, 1.644) | 1.474 (1.295, 1.676) | 1.477 (1.292, 1.671) | 1.477 (1.311, 1.68) | 1.476 (1.302, 1.669) | 1.473 (1.287, 1.669) | 1.477 (1.302, 1.666) | 1.477 (1.302, 1.666) | 1.477 (1.302, 1.666) | 1.477 (1.30, 1.666) |
|  | High exposure | Both | Females | 1.473 (1.287, 1.682) | 1.476 (1.303, 1.681) | 1.469 (1.288, 1.67) | 1.467 (1.282, 1.67) | 1.473 (1.288, 1.66) | 1.475 (1.294, 1.68) | 1.477 (1.3, 1.679) | 1.475 (1.302, 1.674) | 1.476 (1.292, 1.68) | 1.481 (1.309, 1.681) | 1.478 (1.295, 1.683) | 1.473 (1.291, 1.661) | 1.473 (1.29, 1.676) | 1.476 (1.289, 1.662) | 1.476 (1.289, 1.662) | 1.476 (1.289, 1.662) | 1.476 (1.28, 1.662) |
|  | Low exposure | Both | Males | 1.0 (1.0, 1.0) | 1.0 (1.0, 1.0) | 1.0 (1.0, 1.0) | 1.0 (1.0, 1.0) | 1.0 (1.0, 1.0) | 1.0 (1.0, 1.0) | 1.0 (1.0, 1.0) | 1.0 (1.0, 1.0) | 1.0 (1.0, 1.0) | 1.0 (1.0, 1.0) | 1.0 (1.0, 1.0) | 1.0 (1.0, 1.0) | 1.0 (1.0, 1.0) | 1.0 (1.0, 1.0) | 1.0 (1.0, 1.0) | 1.0 (1.0, 1.0) | 1.0 (1.0, 1.0) |
|  | Low exposure | Both | Females | 1.0 (1.0, 1.0) | 1.0 (1.0, 1.0) | 1.0 (1.0, 1.0) | 1.0 (1.0, 1.0) | 1.0 (1.0, 1.0) | 1.0 (1.0, 1.0) | 1.0 (1.0, 1.0) | 1.0 (1.0, 1.0) | 1.0 (1.0, 1.0) | 1.0 (1.0, 1.0) | 1.0 (1.0, 1.0) | 1.0 (1.0, 1.0) | 1.0 (1.0, 1.0) | 1.0 (1.0, 1.0) | 1.0 (1.0, 1.0) | 1.0 (1.0, 1.0) | 1.0 (1.0, 1.0) |
|  | No exposure | Both | Males | 1.0 (1.0, 1.0) | 1.0 (1.0, 1.0) | 1.0 (1.0, 1.0) | 1.0 (1.0, 1.0) | 1.0 (1.0, 1.0) | 1.0 (1.0, 1.0) | 1.0 (1.0, 1.0) | 1.0 (1.0, 1.0) | 1.0 (1.0, 1.0) | 1.0 (1.0, 1.0) | 1.0 (1.0, 1.0) | 1.0 (1.0, 1.0) | 1.0 (1.0, 1.0) | 1.0 (1.0, 1.0) | 1.0 (1.0, 1.0) | 1.0 (1.0, 1.0) | 1.0 (1.0, 1.0) |
|  | No exposure | Both | Females | 1.0 (1.0, 1.0) | 1.0 (1.0, 1.0) | 1.0 (1.0, 1.0) | 1.0 (1.0, 1.0) | 1.0 (1.0, 1.0) | 1.0 (1.0, 1.0) | 1.0 (1.0, 1.0) | 1.0 (1.0, 1.0) | 1.0 (1.0, 1.0) | 1.0 (1.0, 1.0) | 1.0 (1.0, 1.0) | 1.0 (1.0, 1.0) | 1.0 (1.0, 1.0) | 1.0 (1.0, 1.0) | 1.0 (1.0, 1.0) | 1.0 (1.0, 1.0) | 1.0 (1.0, 1.0) |
| **Occupational exposure to formaldehyde** | | | | | | | | | | | | | | | | | | | | |
| Nasopharynx cancer | High exposure | Both | Males | 2.222 (1.026, 4.233) | 2.294 (1.056, 4.409) | 2.204 (1.023, 4.036) | 2.211 (0.994, 4.221) | 2.23 (1.042, 4.13) | 2.241 (1.077, 4.068) | 2.265 (1.056, 4.33) | 2.198 (1.041, 4.081) | 2.232 (1.104, 4.091) | 2.215 (1.022, 4.191) | 2.251 (1.056, 4.236) | 2.233 (1.022, 4.329) | 2.234 (1.02, 4.325) | 2.204 (1.064, 4.14) | 2.204 (1.064, 4.14) | 2.204 (1.064, 4.14) | 2.204 (1.064, 4.14) |
|  | High exposure | Both | Females | 2.202 (1.04, 4.059) | 2.246 (1.039, 4.184) | 2.227 (1.05, 4.207) | 2.269 (1.084, 4.182) | 2.264 (1.069, 4.385) | 2.217 (1.041, 4.21) | 2.201 (1.035, 4.162) | 2.221 (1.035, 4.234) | 2.225 (1.025, 4.212) | 2.276 (1.072, 4.204) | 2.261 (1.04, 4.208) | 2.258 (1.042, 4.371) | 2.241 (1.031, 4.156) | 2.229 (1.025, 4.237) | 2.229 (1.025, 4.237) | 2.229 (1.025, 4.237) | 2.229 (1.02, 4.237) |
|  | Low exposure | Both | Males | 1.0 (1.0, 1.0) | 1.0 (1.0, 1.0) | 1.0 (1.0, 1.0) | 1.0 (1.0, 1.0) | 1.0 (1.0, 1.0) | 1.0 (1.0, 1.0) | 1.0 (1.0, 1.0) | 1.0 (1.0, 1.0) | 1.0 (1.0, 1.0) | 1.0 (1.0, 1.0) | 1.0 (1.0, 1.0) | 1.0 (1.0, 1.0) | 1.0 (1.0, 1.0) | 1.0 (1.0, 1.0) | 1.0 (1.0, 1.0) | 1.0 (1.0, 1.0) | 1.0 (1.0, 1.0) |
|  | Low exposure | Both | Females | 1.0 (1.0, 1.0) | 1.0 (1.0, 1.0) | 1.0 (1.0, 1.0) | 1.0 (1.0, 1.0) | 1.0 (1.0, 1.0) | 1.0 (1.0, 1.0) | 1.0 (1.0, 1.0) | 1.0 (1.0, 1.0) | 1.0 (1.0, 1.0) | 1.0 (1.0, 1.0) | 1.0 (1.0, 1.0) | 1.0 (1.0, 1.0) | 1.0 (1.0, 1.0) | 1.0 (1.0, 1.0) | 1.0 (1.0, 1.0) | 1.0 (1.0, 1.0) | 1.0 (1.0, 1.0) |
|  | No exposure | Both | Males | 1.0 (1.0, 1.0) | 1.0 (1.0, 1.0) | 1.0 (1.0, 1.0) | 1.0 (1.0, 1.0) | 1.0 (1.0, 1.0) | 1.0 (1.0, 1.0) | 1.0 (1.0, 1.0) | 1.0 (1.0, 1.0) | 1.0 (1.0, 1.0) | 1.0 (1.0, 1.0) | 1.0 (1.0, 1.0) | 1.0 (1.0, 1.0) | 1.0 (1.0, 1.0) | 1.0 (1.0, 1.0) | 1.0 (1.0, 1.0) | 1.0 (1.0, 1.0) | 1.0 (1.0, 1.0) |
|  | No exposure | Both | Females | 1.0 (1.0, 1.0) | 1.0 (1.0, 1.0) | 1.0 (1.0, 1.0) | 1.0 (1.0, 1.0) | 1.0 (1.0, 1.0) | 1.0 (1.0, 1.0) | 1.0 (1.0, 1.0) | 1.0 (1.0, 1.0) | 1.0 (1.0, 1.0) | 1.0 (1.0, 1.0) | 1.0 (1.0, 1.0) | 1.0 (1.0, 1.0) | 1.0 (1.0, 1.0) | 1.0 (1.0, 1.0) | 1.0 (1.0, 1.0) | 1.0 (1.0, 1.0) | 1.0 (1.0, 1.0) |
| Acute lymphoid leukemia | High exposure | Both | Males | 1.483 (1.191, 1.818) | 1.479 (1.183, 1.83) | 1.474 (1.182, 1.815) | 1.48 (1.193, 1.827) | 1.479 (1.197, 1.804) | 1.467 (1.174, 1.843) | 1.481 (1.178, 1.839) | 1.49 (1.199, 1.831) | 1.47 (1.197, 1.842) | 1.487 (1.2, 1.846) | 1.485 (1.188, 1.803) | 1.48 (1.18, 1.836) | 1.482 (1.202, 1.839) | 1.488 (1.198, 1.827) | 1.488 (1.198, 1.827) | 1.488 (1.198, 1.827) | 1.488 (1.19, 1.827) |
|  | High exposure | Both | Females | 1.485 (1.199, 1.845) | 1.485 (1.183, 1.855) | 1.479 (1.184, 1.844) | 1.471 (1.181, 1.774) | 1.469 (1.183, 1.819) | 1.486 (1.196, 1.823) | 1.485 (1.19, 1.848) | 1.481 (1.191, 1.856) | 1.48 (1.199, 1.814) | 1.47 (1.184, 1.812) | 1.473 (1.19, 1.814) | 1.471 (1.194, 1.789) | 1.49 (1.209, 1.859) | 1.464 (1.18, 1.788) | 1.464 (1.18, 1.788) | 1.464 (1.18, 1.788) | 1.464 (1.18, 1.788) |
|  | Low exposure | Both | Males | 1.0 (1.0, 1.0) | 1.0 (1.0, 1.0) | 1.0 (1.0, 1.0) | 1.0 (1.0, 1.0) | 1.0 (1.0, 1.0) | 1.0 (1.0, 1.0) | 1.0 (1.0, 1.0) | 1.0 (1.0, 1.0) | 1.0 (1.0, 1.0) | 1.0 (1.0, 1.0) | 1.0 (1.0, 1.0) | 1.0 (1.0, 1.0) | 1.0 (1.0, 1.0) | 1.0 (1.0, 1.0) | 1.0 (1.0, 1.0) | 1.0 (1.0, 1.0) | 1.0 (1.0, 1.0) |
|  | Low exposure | Both | Females | 1.0 (1.0, 1.0) | 1.0 (1.0, 1.0) | 1.0 (1.0, 1.0) | 1.0 (1.0, 1.0) | 1.0 (1.0, 1.0) | 1.0 (1.0, 1.0) | 1.0 (1.0, 1.0) | 1.0 (1.0, 1.0) | 1.0 (1.0, 1.0) | 1.0 (1.0, 1.0) | 1.0 (1.0, 1.0) | 1.0 (1.0, 1.0) | 1.0 (1.0, 1.0) | 1.0 (1.0, 1.0) | 1.0 (1.0, 1.0) | 1.0 (1.0, 1.0) | 1.0 (1.0, 1.0) |
|  | No exposure | Both | Males | 1.0 (1.0, 1.0) | 1.0 (1.0, 1.0) | 1.0 (1.0, 1.0) | 1.0 (1.0, 1.0) | 1.0 (1.0, 1.0) | 1.0 (1.0, 1.0) | 1.0 (1.0, 1.0) | 1.0 (1.0, 1.0) | 1.0 (1.0, 1.0) | 1.0 (1.0, 1.0) | 1.0 (1.0, 1.0) | 1.0 (1.0, 1.0) | 1.0 (1.0, 1.0) | 1.0 (1.0, 1.0) | 1.0 (1.0, 1.0) | 1.0 (1.0, 1.0) | 1.0 (1.0, 1.0) |
|  | No exposure | Both | Females | 1.0 (1.0, 1.0) | 1.0 (1.0, 1.0) | 1.0 (1.0, 1.0) | 1.0 (1.0, 1.0) | 1.0 (1.0, 1.0) | 1.0 (1.0, 1.0) | 1.0 (1.0, 1.0) | 1.0 (1.0, 1.0) | 1.0 (1.0, 1.0) | 1.0 (1.0, 1.0) | 1.0 (1.0, 1.0) | 1.0 (1.0, 1.0) | 1.0 (1.0, 1.0) | 1.0 (1.0, 1.0) | 1.0 (1.0, 1.0) | 1.0 (1.0, 1.0) | 1.0 (1.0, 1.0) |
| Chronic lymphoid leukemia | High exposure | Both | Males | 1.483 (1.191, 1.818) | 1.479 (1.183, 1.83) | 1.474 (1.182, 1.815) | 1.48 (1.193, 1.827) | 1.479 (1.197, 1.804) | 1.467 (1.174, 1.843) | 1.481 (1.178, 1.839) | 1.49 (1.199, 1.831) | 1.47 (1.197, 1.842) | 1.487 (1.2, 1.846) | 1.485 (1.188, 1.803) | 1.48 (1.18, 1.836) | 1.482 (1.202, 1.839) | 1.488 (1.198, 1.827) | 1.488 (1.198, 1.827) | 1.488 (1.198, 1.827) | 1.488 (1.19, 1.827) |
|  | High exposure | Both | Females | 1.485 (1.199, 1.845) | 1.485 (1.183, 1.855) | 1.479 (1.184, 1.844) | 1.471 (1.181, 1.774) | 1.469 (1.183, 1.819) | 1.486 (1.196, 1.823) | 1.485 (1.19, 1.848) | 1.481 (1.191, 1.856) | 1.48 (1.199, 1.814) | 1.47 (1.184, 1.812) | 1.473 (1.19, 1.814) | 1.471 (1.194, 1.789) | 1.49 (1.209, 1.859) | 1.464 (1.18, 1.788) | 1.464 (1.18, 1.788) | 1.464 (1.18, 1.788) | 1.464 (1.18, 1.788) |
|  | Low exposure | Both | Males | 1.0 (1.0, 1.0) | 1.0 (1.0, 1.0) | 1.0 (1.0, 1.0) | 1.0 (1.0, 1.0) | 1.0 (1.0, 1.0) | 1.0 (1.0, 1.0) | 1.0 (1.0, 1.0) | 1.0 (1.0, 1.0) | 1.0 (1.0, 1.0) | 1.0 (1.0, 1.0) | 1.0 (1.0, 1.0) | 1.0 (1.0, 1.0) | 1.0 (1.0, 1.0) | 1.0 (1.0, 1.0) | 1.0 (1.0, 1.0) | 1.0 (1.0, 1.0) | 1.0 (1.0, 1.0) |
|  | Low exposure | Both | Females | 1.0 (1.0, 1.0) | 1.0 (1.0, 1.0) | 1.0 (1.0, 1.0) | 1.0 (1.0, 1.0) | 1.0 (1.0, 1.0) | 1.0 (1.0, 1.0) | 1.0 (1.0, 1.0) | 1.0 (1.0, 1.0) | 1.0 (1.0, 1.0) | 1.0 (1.0, 1.0) | 1.0 (1.0, 1.0) | 1.0 (1.0, 1.0) | 1.0 (1.0, 1.0) | 1.0 (1.0, 1.0) | 1.0 (1.0, 1.0) | 1.0 (1.0, 1.0) | 1.0 (1.0, 1.0) |
|  | No exposure | Both | Males | 1.0 (1.0, 1.0) | 1.0 (1.0, 1.0) | 1.0 (1.0, 1.0) | 1.0 (1.0, 1.0) | 1.0 (1.0, 1.0) | 1.0 (1.0, 1.0) | 1.0 (1.0, 1.0) | 1.0 (1.0, 1.0) | 1.0 (1.0, 1.0) | 1.0 (1.0, 1.0) | 1.0 (1.0, 1.0) | 1.0 (1.0, 1.0) | 1.0 (1.0, 1.0) | 1.0 (1.0, 1.0) | 1.0 (1.0, 1.0) | 1.0 (1.0, 1.0) | 1.0 (1.0, 1.0) |
|  | No exposure | Both | Females | 1.0 (1.0, 1.0) | 1.0 (1.0, 1.0) | 1.0 (1.0, 1.0) | 1.0 (1.0, 1.0) | 1.0 (1.0, 1.0) | 1.0 (1.0, 1.0) | 1.0 (1.0, 1.0) | 1.0 (1.0, 1.0) | 1.0 (1.0, 1.0) | 1.0 (1.0, 1.0) | 1.0 (1.0, 1.0) | 1.0 (1.0, 1.0) | 1.0 (1.0, 1.0) | 1.0 (1.0, 1.0) | 1.0 (1.0, 1.0) | 1.0 (1.0, 1.0) | 1.0 (1.0, 1.0) |
| Acute myeloid leukemia | High exposure | Both | Males | 1.483 (1.191, 1.818) | 1.479 (1.183, 1.83) | 1.474 (1.182, 1.815) | 1.48 (1.193, 1.827) | 1.479 (1.197, 1.804) | 1.467 (1.174, 1.843) | 1.481 (1.178, 1.839) | 1.49 (1.199, 1.831) | 1.47 (1.197, 1.842) | 1.487 (1.2, 1.846) | 1.485 (1.188, 1.803) | 1.48 (1.18, 1.836) | 1.482 (1.202, 1.839) | 1.488 (1.198, 1.827) | 1.488 (1.198, 1.827) | 1.488 (1.198, 1.827) | 1.488 (1.19, 1.827) |
|  | High exposure | Both | Females | 1.485 (1.199, 1.845) | 1.485 (1.183, 1.855) | 1.479 (1.184, 1.844) | 1.471 (1.181, 1.774) | 1.469 (1.183, 1.819) | 1.486 (1.196, 1.823) | 1.485 (1.19, 1.848) | 1.481 (1.191, 1.856) | 1.48 (1.199, 1.814) | 1.47 (1.184, 1.812) | 1.473 (1.19, 1.814) | 1.471 (1.194, 1.789) | 1.49 (1.209, 1.859) | 1.464 (1.18, 1.788) | 1.464 (1.18, 1.788) | 1.464 (1.18, 1.788) | 1.464 (1.18, 1.788) |
|  | Low exposure | Both | Males | 1.0 (1.0, 1.0) | 1.0 (1.0, 1.0) | 1.0 (1.0, 1.0) | 1.0 (1.0, 1.0) | 1.0 (1.0, 1.0) | 1.0 (1.0, 1.0) | 1.0 (1.0, 1.0) | 1.0 (1.0, 1.0) | 1.0 (1.0, 1.0) | 1.0 (1.0, 1.0) | 1.0 (1.0, 1.0) | 1.0 (1.0, 1.0) | 1.0 (1.0, 1.0) | 1.0 (1.0, 1.0) | 1.0 (1.0, 1.0) | 1.0 (1.0, 1.0) | 1.0 (1.0, 1.0) |
|  | Low exposure | Both | Females | 1.0 (1.0, 1.0) | 1.0 (1.0, 1.0) | 1.0 (1.0, 1.0) | 1.0 (1.0, 1.0) | 1.0 (1.0, 1.0) | 1.0 (1.0, 1.0) | 1.0 (1.0, 1.0) | 1.0 (1.0, 1.0) | 1.0 (1.0, 1.0) | 1.0 (1.0, 1.0) | 1.0 (1.0, 1.0) | 1.0 (1.0, 1.0) | 1.0 (1.0, 1.0) | 1.0 (1.0, 1.0) | 1.0 (1.0, 1.0) | 1.0 (1.0, 1.0) | 1.0 (1.0, 1.0) |
|  | No exposure | Both | Males | 1.0 (1.0, 1.0) | 1.0 (1.0, 1.0) | 1.0 (1.0, 1.0) | 1.0 (1.0, 1.0) | 1.0 (1.0, 1.0) | 1.0 (1.0, 1.0) | 1.0 (1.0, 1.0) | 1.0 (1.0, 1.0) | 1.0 (1.0, 1.0) | 1.0 (1.0, 1.0) | 1.0 (1.0, 1.0) | 1.0 (1.0, 1.0) | 1.0 (1.0, 1.0) | 1.0 (1.0, 1.0) | 1.0 (1.0, 1.0) | 1.0 (1.0, 1.0) | 1.0 (1.0, 1.0) |
|  | No exposure | Both | Females | 1.0 (1.0, 1.0) | 1.0 (1.0, 1.0) | 1.0 (1.0, 1.0) | 1.0 (1.0, 1.0) | 1.0 (1.0, 1.0) | 1.0 (1.0, 1.0) | 1.0 (1.0, 1.0) | 1.0 (1.0, 1.0) | 1.0 (1.0, 1.0) | 1.0 (1.0, 1.0) | 1.0 (1.0, 1.0) | 1.0 (1.0, 1.0) | 1.0 (1.0, 1.0) | 1.0 (1.0, 1.0) | 1.0 (1.0, 1.0) | 1.0 (1.0, 1.0) | 1.0 (1.0, 1.0) |
| Chronic myeloid leukaemia | High exposure | Both | Males | 1.483 (1.191, 1.818) | 1.479 (1.183, 1.83) | 1.474 (1.182, 1.815) | 1.48 (1.193, 1.827) | 1.479 (1.197, 1.804) | 1.467 (1.174, 1.843) | 1.481 (1.178, 1.839) | 1.49 (1.199, 1.831) | 1.47 (1.197, 1.842) | 1.487 (1.2, 1.846) | 1.485 (1.188, 1.803) | 1.48 (1.18, 1.836) | 1.482 (1.202, 1.839) | 1.488 (1.198, 1.827) | 1.488 (1.198, 1.827) | 1.488 (1.198, 1.827) | 1.488 (1.19, 1.827) |
|  | High exposure | Both | Females | 1.485 (1.199, 1.845) | 1.485 (1.183, 1.855) | 1.479 (1.184, 1.844) | 1.471 (1.181, 1.774) | 1.469 (1.183, 1.819) | 1.486 (1.196, 1.823) | 1.485 (1.19, 1.848) | 1.481 (1.191, 1.856) | 1.48 (1.199, 1.814) | 1.47 (1.184, 1.812) | 1.473 (1.19, 1.814) | 1.471 (1.194, 1.789) | 1.49 (1.209, 1.859) | 1.464 (1.18, 1.788) | 1.464 (1.18, 1.788) | 1.464 (1.18, 1.788) | 1.464 (1.18, 1.788) |
|  | Low exposure | Both | Males | 1.0 (1.0, 1.0) | 1.0 (1.0, 1.0) | 1.0 (1.0, 1.0) | 1.0 (1.0, 1.0) | 1.0 (1.0, 1.0) | 1.0 (1.0, 1.0) | 1.0 (1.0, 1.0) | 1.0 (1.0, 1.0) | 1.0 (1.0, 1.0) | 1.0 (1.0, 1.0) | 1.0 (1.0, 1.0) | 1.0 (1.0, 1.0) | 1.0 (1.0, 1.0) | 1.0 (1.0, 1.0) | 1.0 (1.0, 1.0) | 1.0 (1.0, 1.0) | 1.0 (1.0, 1.0) |
|  | Low exposure | Both | Females | 1.0 (1.0, 1.0) | 1.0 (1.0, 1.0) | 1.0 (1.0, 1.0) | 1.0 (1.0, 1.0) | 1.0 (1.0, 1.0) | 1.0 (1.0, 1.0) | 1.0 (1.0, 1.0) | 1.0 (1.0, 1.0) | 1.0 (1.0, 1.0) | 1.0 (1.0, 1.0) | 1.0 (1.0, 1.0) | 1.0 (1.0, 1.0) | 1.0 (1.0, 1.0) | 1.0 (1.0, 1.0) | 1.0 (1.0, 1.0) | 1.0 (1.0, 1.0) | 1.0 (1.0, 1.0) |
|  | No exposure | Both | Males | 1.0 (1.0, 1.0) | 1.0 (1.0, 1.0) | 1.0 (1.0, 1.0) | 1.0 (1.0, 1.0) | 1.0 (1.0, 1.0) | 1.0 (1.0, 1.0) | 1.0 (1.0, 1.0) | 1.0 (1.0, 1.0) | 1.0 (1.0, 1.0) | 1.0 (1.0, 1.0) | 1.0 (1.0, 1.0) | 1.0 (1.0, 1.0) | 1.0 (1.0, 1.0) | 1.0 (1.0, 1.0) | 1.0 (1.0, 1.0) | 1.0 (1.0, 1.0) | 1.0 (1.0, 1.0) |
|  | No exposure | Both | Females | 1.0 (1.0, 1.0) | 1.0 (1.0, 1.0) | 1.0 (1.0, 1.0) | 1.0 (1.0, 1.0) | 1.0 (1.0, 1.0) | 1.0 (1.0, 1.0) | 1.0 (1.0, 1.0) | 1.0 (1.0, 1.0) | 1.0 (1.0, 1.0) | 1.0 (1.0, 1.0) | 1.0 (1.0, 1.0) | 1.0 (1.0, 1.0) | 1.0 (1.0, 1.0) | 1.0 (1.0, 1.0) | 1.0 (1.0, 1.0) | 1.0 (1.0, 1.0) | 1.0 (1.0, 1.0) |
| Other leukemia | High exposure | Both | Males | 1.483 (1.191, 1.818) | 1.479 (1.183, 1.83) | 1.474 (1.182, 1.815) | 1.48 (1.193, 1.827) | 1.479 (1.197, 1.804) | 1.467 (1.174, 1.843) | 1.481 (1.178, 1.839) | 1.49 (1.199, 1.831) | 1.47 (1.197, 1.842) | 1.487 (1.2, 1.846) | 1.485 (1.188, 1.803) | 1.48 (1.18, 1.836) | 1.482 (1.202, 1.839) | 1.488 (1.198, 1.827) | 1.488 (1.198, 1.827) | 1.488 (1.198, 1.827) | 1.488 (1.19, 1.827) |
|  | High exposure | Both | Females | 1.485 (1.199, 1.845) | 1.485 (1.183, 1.855) | 1.479 (1.184, 1.844) | 1.471 (1.181, 1.774) | 1.469 (1.183, 1.819) | 1.486 (1.196, 1.823) | 1.485 (1.19, 1.848) | 1.481 (1.191, 1.856) | 1.48 (1.199, 1.814) | 1.47 (1.184, 1.812) | 1.473 (1.19, 1.814) | 1.471 (1.194, 1.789) | 1.49 (1.209, 1.859) | 1.464 (1.18, 1.788) | 1.464 (1.18, 1.788) | 1.464 (1.18, 1.788) | 1.464 (1.18, 1.788) |
|  | Low exposure | Both | Males | 1.0 (1.0, 1.0) | 1.0 (1.0, 1.0) | 1.0 (1.0, 1.0) | 1.0 (1.0, 1.0) | 1.0 (1.0, 1.0) | 1.0 (1.0, 1.0) | 1.0 (1.0, 1.0) | 1.0 (1.0, 1.0) | 1.0 (1.0, 1.0) | 1.0 (1.0, 1.0) | 1.0 (1.0, 1.0) | 1.0 (1.0, 1.0) | 1.0 (1.0, 1.0) | 1.0 (1.0, 1.0) | 1.0 (1.0, 1.0) | 1.0 (1.0, 1.0) | 1.0 (1.0, 1.0) |
|  | Low exposure | Both | Females | 1.0 (1.0, 1.0) | 1.0 (1.0, 1.0) | 1.0 (1.0, 1.0) | 1.0 (1.0, 1.0) | 1.0 (1.0, 1.0) | 1.0 (1.0, 1.0) | 1.0 (1.0, 1.0) | 1.0 (1.0, 1.0) | 1.0 (1.0, 1.0) | 1.0 (1.0, 1.0) | 1.0 (1.0, 1.0) | 1.0 (1.0, 1.0) | 1.0 (1.0, 1.0) | 1.0 (1.0, 1.0) | 1.0 (1.0, 1.0) | 1.0 (1.0, 1.0) | 1.0 (1.0, 1.0) |
|  | No exposure | Both | Males | 1.0 (1.0, 1.0) | 1.0 (1.0, 1.0) | 1.0 (1.0, 1.0) | 1.0 (1.0, 1.0) | 1.0 (1.0, 1.0) | 1.0 (1.0, 1.0) | 1.0 (1.0, 1.0) | 1.0 (1.0, 1.0) | 1.0 (1.0, 1.0) | 1.0 (1.0, 1.0) | 1.0 (1.0, 1.0) | 1.0 (1.0, 1.0) | 1.0 (1.0, 1.0) | 1.0 (1.0, 1.0) | 1.0 (1.0, 1.0) | 1.0 (1.0, 1.0) | 1.0 (1.0, 1.0) |
|  | No exposure | Both | Females | 1.0 (1.0, 1.0) | 1.0 (1.0, 1.0) | 1.0 (1.0, 1.0) | 1.0 (1.0, 1.0) | 1.0 (1.0, 1.0) | 1.0 (1.0, 1.0) | 1.0 (1.0, 1.0) | 1.0 (1.0, 1.0) | 1.0 (1.0, 1.0) | 1.0 (1.0, 1.0) | 1.0 (1.0, 1.0) | 1.0 (1.0, 1.0) | 1.0 (1.0, 1.0) | 1.0 (1.0, 1.0) | 1.0 (1.0, 1.0) | 1.0 (1.0, 1.0) | 1.0 (1.0, 1.0) |
| **Occupational exposure to nickel** | | | | | | | | | | | | | | | | | | | | |
| Tracheal, bronchus, and lung cancer | High exposure | Both | Both | 2.148 (1.296, 3.297) | 2.148 (1.296, 3.297) | 2.148 (1.296, 3.297) | 2.148 (1.296, 3.297) | 2.148 (1.296, 3.297) | 2.148 (1.296, 3.297) | 2.148 (1.296, 3.297) | 2.148 (1.296, 3.297) | 2.148 (1.296, 3.297) | 2.148 (1.296, 3.297) | 2.148 (1.296, 3.297) | 2.148 (1.296, 3.297) | 2.148 (1.296, 3.297) | 2.148 (1.296, 3.297) | 2.148 (1.296, 3.297) | 2.148 (1.296, 3.297) | 2.148 (1.29, 3.297) |
|  | Low exposure | Both | Both | 1.538 (0.606, 3.355) | 1.538 (0.606, 3.355) | 1.538 (0.606, 3.355) | 1.538 (0.606, 3.355) | 1.538 (0.606, 3.355) | 1.538 (0.606, 3.355) | 1.538 (0.606, 3.355) | 1.538 (0.606, 3.355) | 1.538 (0.606, 3.355) | 1.538 (0.606, 3.355) | 1.538 (0.606, 3.355) | 1.538 (0.606, 3.355) | 1.538 (0.606, 3.355) | 1.538 (0.606, 3.355) | 1.538 (0.606, 3.355) | 1.538 (0.606, 3.355) | 1.538 (0.60, 3.355) |
|  | No exposure | Both | Both | 1.0 (1.0, 1.0) | 1.0 (1.0, 1.0) | 1.0 (1.0, 1.0) | 1.0 (1.0, 1.0) | 1.0 (1.0, 1.0) | 1.0 (1.0, 1.0) | 1.0 (1.0, 1.0) | 1.0 (1.0, 1.0) | 1.0 (1.0, 1.0) | 1.0 (1.0, 1.0) | 1.0 (1.0, 1.0) | 1.0 (1.0, 1.0) | 1.0 (1.0, 1.0) | 1.0 (1.0, 1.0) | 1.0 (1.0, 1.0) | 1.0 (1.0, 1.0) | 1.0 (1.0, 1.0) |
| **Occupational exposure to polycyclic aromatic hydrocarbons** | | | | | | | | | | | | | | | | | | | | |
| Tracheal, bronchus, and lung cancer | High exposure | Both | Males | 1.31 (1.165, 1.468) | 1.304 (1.146, 1.466) | 1.313 (1.164, 1.485) | 1.314 (1.167, 1.477) | 1.315 (1.17, 1.474) | 1.314 (1.16, 1.475) | 1.315 (1.154, 1.477) | 1.312 (1.16, 1.477) | 1.318 (1.166, 1.483) | 1.311 (1.158, 1.479) | 1.313 (1.166, 1.485) | 1.314 (1.154, 1.483) | 1.313 (1.161, 1.478) | 1.309 (1.176, 1.477) | 1.309 (1.176, 1.477) | 1.309 (1.176, 1.477) | 1.309 (1.17, 1.477) |
|  | High exposure | Both | Females | 1.31 (1.154, 1.486) | 1.311 (1.157, 1.469) | 1.313 (1.154, 1.472) | 1.313 (1.155, 1.469) | 1.316 (1.162, 1.483) | 1.313 (1.156, 1.476) | 1.314 (1.155, 1.483) | 1.314 (1.171, 1.483) | 1.311 (1.146, 1.477) | 1.312 (1.163, 1.481) | 1.309 (1.147, 1.489) | 1.316 (1.168, 1.475) | 1.315 (1.15, 1.479) | 1.315 (1.166, 1.481) | 1.315 (1.166, 1.481) | 1.315 (1.166, 1.481) | 1.315 (1.16, 1.481) |
|  | Low exposure | Both | Males | 1.0 (1.0, 1.0) | 1.0 (1.0, 1.0) | 1.0 (1.0, 1.0) | 1.0 (1.0, 1.0) | 1.0 (1.0, 1.0) | 1.0 (1.0, 1.0) | 1.0 (1.0, 1.0) | 1.0 (1.0, 1.0) | 1.0 (1.0, 1.0) | 1.0 (1.0, 1.0) | 1.0 (1.0, 1.0) | 1.0 (1.0, 1.0) | 1.0 (1.0, 1.0) | 1.0 (1.0, 1.0) | 1.0 (1.0, 1.0) | 1.0 (1.0, 1.0) | 1.0 (1.0, 1.0) |
|  | Low exposure | Both | Females | 1.0 (1.0, 1.0) | 1.0 (1.0, 1.0) | 1.0 (1.0, 1.0) | 1.0 (1.0, 1.0) | 1.0 (1.0, 1.0) | 1.0 (1.0, 1.0) | 1.0 (1.0, 1.0) | 1.0 (1.0, 1.0) | 1.0 (1.0, 1.0) | 1.0 (1.0, 1.0) | 1.0 (1.0, 1.0) | 1.0 (1.0, 1.0) | 1.0 (1.0, 1.0) | 1.0 (1.0, 1.0) | 1.0 (1.0, 1.0) | 1.0 (1.0, 1.0) | 1.0 (1.0, 1.0) |
|  | No exposure | Both | Males | 1.0 (1.0, 1.0) | 1.0 (1.0, 1.0) | 1.0 (1.0, 1.0) | 1.0 (1.0, 1.0) | 1.0 (1.0, 1.0) | 1.0 (1.0, 1.0) | 1.0 (1.0, 1.0) | 1.0 (1.0, 1.0) | 1.0 (1.0, 1.0) | 1.0 (1.0, 1.0) | 1.0 (1.0, 1.0) | 1.0 (1.0, 1.0) | 1.0 (1.0, 1.0) | 1.0 (1.0, 1.0) | 1.0 (1.0, 1.0) | 1.0 (1.0, 1.0) | 1.0 (1.0, 1.0) |
|  | No exposure | Both | Females | 1.0 (1.0, 1.0) | 1.0 (1.0, 1.0) | 1.0 (1.0, 1.0) | 1.0 (1.0, 1.0) | 1.0 (1.0, 1.0) | 1.0 (1.0, 1.0) | 1.0 (1.0, 1.0) | 1.0 (1.0, 1.0) | 1.0 (1.0, 1.0) | 1.0 (1.0, 1.0) | 1.0 (1.0, 1.0) | 1.0 (1.0, 1.0) | 1.0 (1.0, 1.0) | 1.0 (1.0, 1.0) | 1.0 (1.0, 1.0) | 1.0 (1.0, 1.0) | 1.0 (1.0, 1.0) |
| **Occupational exposure to silica** | | | | | | | | | | | | | | | | | | | | |
| Tracheal, bronchus, and lung cancer | High exposure | Both | Both | 1.698 (1.164, 2.259) | 1.698 (1.164, 2.259) | 1.698 (1.164, 2.259) | 1.698 (1.164, 2.259) | 1.698 (1.164, 2.259) | 1.698 (1.164, 2.259) | 1.698 (1.164, 2.259) | 1.698 (1.164, 2.259) | 1.698 (1.164, 2.259) | 1.698 (1.164, 2.259) | 1.698 (1.164, 2.259) | 1.698 (1.164, 2.259) | 1.698 (1.164, 2.259) | 1.698 (1.164, 2.259) | 1.698 (1.164, 2.259) | 1.698 (1.164, 2.259) | 1.698 (1.16, 2.259) |
|  | Low exposure | Both | Both | 1.537 (1.063, 1.986) | 1.537 (1.063, 1.986) | 1.537 (1.063, 1.986) | 1.537 (1.063, 1.986) | 1.537 (1.063, 1.986) | 1.537 (1.063, 1.986) | 1.537 (1.063, 1.986) | 1.537 (1.063, 1.986) | 1.537 (1.063, 1.986) | 1.537 (1.063, 1.986) | 1.537 (1.063, 1.986) | 1.537 (1.063, 1.986) | 1.537 (1.063, 1.986) | 1.537 (1.063, 1.986) | 1.537 (1.063, 1.986) | 1.537 (1.063, 1.986) | 1.537 (1.06, 1.986) |
|  | No exposure | Both | Both | 1.0 (1.0, 1.0) | 1.0 (1.0, 1.0) | 1.0 (1.0, 1.0) | 1.0 (1.0, 1.0) | 1.0 (1.0, 1.0) | 1.0 (1.0, 1.0) | 1.0 (1.0, 1.0) | 1.0 (1.0, 1.0) | 1.0 (1.0, 1.0) | 1.0 (1.0, 1.0) | 1.0 (1.0, 1.0) | 1.0 (1.0, 1.0) | 1.0 (1.0, 1.0) | 1.0 (1.0, 1.0) | 1.0 (1.0, 1.0) | 1.0 (1.0, 1.0) | 1.0 (1.0, 1.0) |
| **Occupational exposure to sulfuric acid** | | | | | | | | | | | | | | | | | | | | |
| Larynx cancer | High exposure | Both | Both | 4.566 (2.122, 8.328) | 4.566 (2.122, 8.328) | 4.566 (2.122, 8.328) | 4.566 (2.122, 8.328) | 4.566 (2.122, 8.328) | 4.566 (2.122, 8.328) | 4.566 (2.122, 8.328) | 4.566 (2.122, 8.328) | 4.566 (2.122, 8.328) | 4.566 (2.122, 8.328) | 4.566 (2.122, 8.328) | 4.566 (2.122, 8.328) | 4.566 (2.122, 8.328) | 4.566 (2.122, 8.328) | 4.566 (2.122, 8.328) | 4.566 (2.122, 8.328) | 4.566 (2.12, 8.328) |
|  | Low exposure | Both | Both | 2.024 (0.944, 3.782) | 2.024 (0.944, 3.782) | 2.024 (0.944, 3.782) | 2.024 (0.944, 3.782) | 2.024 (0.944, 3.782) | 2.024 (0.944, 3.782) | 2.024 (0.944, 3.782) | 2.024 (0.944, 3.782) | 2.024 (0.944, 3.782) | 2.024 (0.944, 3.782) | 2.024 (0.944, 3.782) | 2.024 (0.944, 3.782) | 2.024 (0.944, 3.782) | 2.024 (0.944, 3.782) | 2.024 (0.944, 3.782) | 2.024 (0.944, 3.782) | 2.024 (0.94, 3.782) |
|  | No exposure | Both | Both | 1.0 (1.0, 1.0) | 1.0 (1.0, 1.0) | 1.0 (1.0, 1.0) | 1.0 (1.0, 1.0) | 1.0 (1.0, 1.0) | 1.0 (1.0, 1.0) | 1.0 (1.0, 1.0) | 1.0 (1.0, 1.0) | 1.0 (1.0, 1.0) | 1.0 (1.0, 1.0) | 1.0 (1.0, 1.0) | 1.0 (1.0, 1.0) | 1.0 (1.0, 1.0) | 1.0 (1.0, 1.0) | 1.0 (1.0, 1.0) | 1.0 (1.0, 1.0) | 1.0 (1.0, 1.0) |
| **Occupational exposure to trichloroethylene** | | | | | | | | | | | | | | | | | | | | |
| Kidney cancer | High exposure | Both | Both | 1.245 (1.054, 1.456) | 1.245 (1.054, 1.456) | 1.245 (1.054, 1.456) | 1.245 (1.054, 1.456) | 1.245 (1.054, 1.456) | 1.245 (1.054, 1.456) | 1.245 (1.054, 1.456) | 1.245 (1.054, 1.456) | 1.245 (1.054, 1.456) | 1.245 (1.054, 1.456) | 1.245 (1.054, 1.456) | 1.245 (1.054, 1.456) | 1.245 (1.054, 1.456) | 1.245 (1.054, 1.456) | 1.245 (1.054, 1.456) | 1.245 (1.054, 1.456) | 1.245 (1.05, 1.456) |
|  | Low exposure | Both | Both | 1.0 (1.0, 1.0) | 1.0 (1.0, 1.0) | 1.0 (1.0, 1.0) | 1.0 (1.0, 1.0) | 1.0 (1.0, 1.0) | 1.0 (1.0, 1.0) | 1.0 (1.0, 1.0) | 1.0 (1.0, 1.0) | 1.0 (1.0, 1.0) | 1.0 (1.0, 1.0) | 1.0 (1.0, 1.0) | 1.0 (1.0, 1.0) | 1.0 (1.0, 1.0) | 1.0 (1.0, 1.0) | 1.0 (1.0, 1.0) | 1.0 (1.0, 1.0) | 1.0 (1.0, 1.0) |
|  | No exposure | Both | Both | 1.0 (1.0, 1.0) | 1.0 (1.0, 1.0) | 1.0 (1.0, 1.0) | 1.0 (1.0, 1.0) | 1.0 (1.0, 1.0) | 1.0 (1.0, 1.0) | 1.0 (1.0, 1.0) | 1.0 (1.0, 1.0) | 1.0 (1.0, 1.0) | 1.0 (1.0, 1.0) | 1.0 (1.0, 1.0) | 1.0 (1.0, 1.0) | 1.0 (1.0, 1.0) | 1.0 (1.0, 1.0) | 1.0 (1.0, 1.0) | 1.0 (1.0, 1.0) | 1.0 (1.0, 1.0) |
| **Occupational asthmagens** | | | | | | | | | | | | | | | | | | | | |
| Asthma | Admin | Both | Males | 1.0 (1.0, 1.0) | 1.0 (1.0, 1.0) | 1.0 (1.0, 1.0) | 1.0 (1.0, 1.0) | 1.0 (1.0, 1.0) | 1.0 (1.0, 1.0) | 1.0 (1.0, 1.0) | 1.0 (1.0, 1.0) | 1.0 (1.0, 1.0) | 1.0 (1.0, 1.0) | 1.0 (1.0, 1.0) | 1.0 (1.0, 1.0) | 1.0 (1.0, 1.0) | 1.0 (1.0, 1.0) |  |  |  |
|  | Admin | Both | Females | 1.0 (1.0, 1.0) | 1.0 (1.0, 1.0) | 1.0 (1.0, 1.0) | 1.0 (1.0, 1.0) | 1.0 (1.0, 1.0) | 1.0 (1.0, 1.0) | 1.0 (1.0, 1.0) | 1.0 (1.0, 1.0) | 1.0 (1.0, 1.0) | 1.0 (1.0, 1.0) | 1.0 (1.0, 1.0) | 1.0 (1.0, 1.0) | 1.0 (1.0, 1.0) | 1.0 (1.0, 1.0) |  |  |  |
|  | Technical | Both | Males | 1.05 (0.977, 1.125) | 1.051 (0.979, 1.122) | 1.051 (0.982, 1.124) | 1.051 (0.983, 1.118) | 1.05 (0.977, 1.12) | 1.05 (0.981, 1.122) | 1.051 (0.98, 1.121) | 1.051 (0.986, 1.121) | 1.05 (0.976, 1.125) | 1.05 (0.987, 1.118) | 1.051 (0.984, 1.121) | 1.051 (0.982, 1.122) | 1.051 (0.981, 1.122) | 1.051 (0.981, 1.122) |  |  |  |
|  | Technical | Both | Females | 1.06 (1.028, 1.095) | 1.06 (1.024, 1.099) | 1.059 (1.025, 1.095) | 1.061 (1.026, 1.099) | 1.06 (1.027, 1.093) | 1.06 (1.026, 1.094) | 1.059 (1.024, 1.093) | 1.06 (1.028, 1.094) | 1.06 (1.027, 1.092) | 1.061 (1.026, 1.095) | 1.06 (1.027, 1.096) | 1.061 (1.023, 1.096) | 1.06 (1.027, 1.096) | 1.06 (1.027, 1.096) |  |  |  |
|  | Sales | Both | Males | 1.14 (1.047, 1.237) | 1.14 (1.049, 1.234) | 1.144 (1.055, 1.233) | 1.142 (1.048, 1.234) | 1.14 (1.046, 1.234) | 1.141 (1.053, 1.235) | 1.143 (1.052, 1.238) | 1.14 (1.047, 1.236) | 1.141 (1.056, 1.235) | 1.138 (1.046, 1.236) | 1.139 (1.057, 1.226) | 1.141 (1.052, 1.243) | 1.14 (1.058, 1.239) | 1.14 (1.058, 1.239) |  |  |  |
|  | Sales | Both | Females | 1.131 (1.083, 1.182) | 1.13 (1.083, 1.178) | 1.129 (1.083, 1.178) | 1.13 (1.081, 1.182) | 1.131 (1.084, 1.18) | 1.13 (1.08, 1.181) | 1.13 (1.083, 1.181) | 1.13 (1.079, 1.178) | 1.131 (1.077, 1.184) | 1.13 (1.081, 1.182) | 1.129 (1.082, 1.181) | 1.131 (1.083, 1.184) | 1.131 (1.083, 1.183) | 1.131 (1.083, 1.183) |  |  |  |
|  | Agriculture | Both | Males | 1.519 (1.1, 2.029) | 1.527 (1.119, 2.063) | 1.524 (1.103, 2.019) | 1.513 (1.127, 1.999) | 1.523 (1.108, 2.023) | 1.516 (1.083, 2.043) | 1.522 (1.106, 2.038) | 1.52 (1.122, 2.023) | 1.519 (1.103, 2.011) | 1.531 (1.122, 2.047) | 1.52 (1.106, 2.041) | 1.508 (1.083, 2.019) | 1.498 (1.081, 1.965) | 1.498 (1.081, 1.965) |  |  |  |
|  | Agriculture | Both | Females | 1.52 (1.125, 2.022) | 1.506 (1.099, 1.997) | 1.52 (1.115, 2.05) | 1.514 (1.109, 2.048) | 1.509 (1.101, 1.969) | 1.513 (1.128, 2.026) | 1.508 (1.108, 2.0) | 1.526 (1.108, 2.07) | 1.519 (1.117, 1.981) | 1.53 (1.105, 2.017) | 1.519 (1.107, 2.024) | 1.518 (1.115, 2.018) | 1.502 (1.094, 2.03) | 1.502 (1.094, 2.03) |  |  |  |
|  | Mining | Both | Males | 1.959 (1.576, 2.413) | 1.959 (1.568, 2.381) | 1.971 (1.602, 2.414) | 1.966 (1.571, 2.396) | 1.963 (1.601, 2.395) | 1.959 (1.588, 2.385) | 1.956 (1.595, 2.406) | 1.964 (1.57, 2.397) | 1.969 (1.616, 2.417) | 1.954 (1.574, 2.406) | 1.965 (1.558, 2.393) | 1.955 (1.577, 2.39) | 1.953 (1.551, 2.418) | 1.953 (1.551, 2.418) |  |  |  |
|  | Mining | Both | Females | 1.956 (1.58, 2.408) | 1.961 (1.574, 2.395) | 1.967 (1.586, 2.417) | 1.952 (1.567, 2.371) | 1.959 (1.575, 2.416) | 1.959 (1.567, 2.417) | 1.962 (1.585, 2.386) | 1.955 (1.591, 2.393) | 1.948 (1.57, 2.381) | 1.964 (1.597, 2.409) | 1.965 (1.59, 2.414) | 1.959 (1.588, 2.39) | 1.973 (1.586, 2.392) | 1.973 (1.586, 2.392) |  |  |  |
|  | Transport | Both | Males | 1.313 (1.22, 1.402) | 1.311 (1.222, 1.406) | 1.312 (1.225, 1.399) | 1.31 (1.218, 1.398) | 1.312 (1.225, 1.404) | 1.311 (1.225, 1.401) | 1.313 (1.226, 1.402) | 1.31 (1.223, 1.398) | 1.314 (1.221, 1.396) | 1.313 (1.224, 1.408) | 1.311 (1.228, 1.397) | 1.312 (1.225, 1.409) | 1.311 (1.226, 1.407) | 1.311 (1.226, 1.407) |  |  |  |
|  | Transport | Both | Females | 1.221 (1.132, 1.312) | 1.22 (1.138, 1.31) | 1.217 (1.136, 1.303) | 1.221 (1.138, 1.313) | 1.221 (1.137, 1.317) | 1.221 (1.132, 1.314) | 1.22 (1.132, 1.312) | 1.22 (1.134, 1.313) | 1.221 (1.14, 1.315) | 1.223 (1.139, 1.309) | 1.221 (1.133, 1.312) | 1.224 (1.135, 1.316) | 1.22 (1.133, 1.313) | 1.22 (1.133, 1.313) |  |  |  |
|  | Manufacture | Both | Males | 1.559 (1.477, 1.647) | 1.561 (1.474, 1.652) | 1.56 (1.474, 1.657) | 1.562 (1.472, 1.658) | 1.561 (1.473, 1.65) | 1.561 (1.471, 1.655) | 1.56 (1.471, 1.649) | 1.562 (1.473, 1.654) | 1.558 (1.461, 1.655) | 1.562 (1.479, 1.655) | 1.559 (1.475, 1.652) | 1.562 (1.476, 1.657) | 1.561 (1.474, 1.654) | 1.561 (1.474, 1.654) |  |  |  |
|  | Manufacture | Both | Females | 1.33 (1.272, 1.392) | 1.331 (1.27, 1.39) | 1.33 (1.271, 1.39) | 1.329 (1.271, 1.388) | 1.331 (1.268, 1.392) | 1.329 (1.269, 1.39) | 1.33 (1.269, 1.391) | 1.332 (1.272, 1.394) | 1.331 (1.273, 1.389) | 1.331 (1.272, 1.391) | 1.33 (1.268, 1.392) | 1.33 (1.268, 1.394) | 1.331 (1.272, 1.39) | 1.331 (1.272, 1.39) |  |  |  |
|  | Services | Both | Males | 1.531 (1.415, 1.646) | 1.531 (1.416, 1.652) | 1.529 (1.409, 1.649) | 1.532 (1.413, 1.653) | 1.529 (1.424, 1.648) | 1.529 (1.416, 1.645) | 1.533 (1.418, 1.646) | 1.535 (1.427, 1.649) | 1.531 (1.416, 1.655) | 1.528 (1.411, 1.651) | 1.53 (1.416, 1.652) | 1.53 (1.411, 1.658) | 1.531 (1.415, 1.655) | 1.531 (1.415, 1.655) |  |  |  |
|  | Services | Both | Females | 1.41 (1.352, 1.467) | 1.41 (1.357, 1.469) | 1.412 (1.357, 1.467) | 1.41 (1.356, 1.464) | 1.409 (1.353, 1.463) | 1.409 (1.354, 1.467) | 1.41 (1.354, 1.467) | 1.411 (1.357, 1.464) | 1.409 (1.357, 1.46) | 1.411 (1.355, 1.466) | 1.41 (1.354, 1.465) | 1.41 (1.357, 1.467) | 1.411 (1.357, 1.464) | 1.411 (1.357, 1.464) |  |  |  |
|  | Other | Both | Males | 1.0 (1.0, 1.0) | 1.0 (1.0, 1.0) | 1.0 (1.0, 1.0) | 1.0 (1.0, 1.0) | 1.0 (1.0, 1.0) | 1.0 (1.0, 1.0) | 1.0 (1.0, 1.0) | 1.0 (1.0, 1.0) | 1.0 (1.0, 1.0) | 1.0 (1.0, 1.0) | 1.0 (1.0, 1.0) | 1.0 (1.0, 1.0) | 1.0 (1.0, 1.0) | 1.0 (1.0, 1.0) |  |  |  |
|  | Other | Both | Females | 1.0 (1.0, 1.0) | 1.0 (1.0, 1.0) | 1.0 (1.0, 1.0) | 1.0 (1.0, 1.0) | 1.0 (1.0, 1.0) | 1.0 (1.0, 1.0) | 1.0 (1.0, 1.0) | 1.0 (1.0, 1.0) | 1.0 (1.0, 1.0) | 1.0 (1.0, 1.0) | 1.0 (1.0, 1.0) | 1.0 (1.0, 1.0) | 1.0 (1.0, 1.0) | 1.0 (1.0, 1.0) |  |  |  |
| **Occupational particulate matter, gases, and fumes** | | | | | | | | | | | | | | | | | | | | |
| Chronic obstructive pulmonary disease | High | Both | Males | 2.364 (1.463, 3.64) | 2.341 (1.426, 3.555) | 2.37 (1.41, 3.596) | 2.391 (1.455, 3.756) | 2.387 (1.409, 3.791) | 2.379 (1.4, 3.672) | 2.409 (1.439, 3.833) | 2.361 (1.447, 3.684) | 2.367 (1.454, 3.654) | 2.373 (1.375, 3.64) | 2.357 (1.438, 3.685) | 2.402 (1.434, 3.763) | 2.398 (1.441, 3.813) | 2.359 (1.429, 3.695) | 2.359 (1.429, 3.695) | 2.359 (1.429, 3.695) | 2.359 (1.42, 3.695) |
|  | High | Both | Females | 2.371 (1.459, 3.719) | 2.395 (1.483, 3.669) | 2.364 (1.473, 3.694) | 2.377 (1.431, 3.763) | 2.375 (1.446, 3.698) | 2.326 (1.438, 3.576) | 2.35 (1.432, 3.702) | 2.35 (1.425, 3.609) | 2.364 (1.461, 3.677) | 2.364 (1.475, 3.64) | 2.395 (1.476, 3.715) | 2.336 (1.434, 3.673) | 2.363 (1.431, 3.741) | 2.404 (1.462, 3.619) | 2.404 (1.462, 3.619) | 2.404 (1.462, 3.619) | 2.404 (1.46, 3.619) |
|  | Low | Both | Males | 1.462 (1.057, 1.915) | 1.457 (1.052, 1.935) | 1.464 (1.085, 1.91) | 1.45 (1.067, 1.947) | 1.452 (1.06, 1.963) | 1.454 (1.075, 1.96) | 1.446 (1.055, 1.929) | 1.457 (1.076, 1.933) | 1.453 (1.052, 1.903) | 1.46 (1.067, 1.954) | 1.443 (1.057, 1.916) | 1.462 (1.097, 1.954) | 1.464 (1.079, 1.952) | 1.462 (1.096, 1.965) | 1.462 (1.096, 1.965) | 1.462 (1.096, 1.965) | 1.462 (1.09, 1.965) |
|  | Low | Both | Females | 1.446 (1.063, 1.904) | 1.459 (1.09, 1.954) | 1.453 (1.055, 2.003) | 1.459 (1.072, 1.931) | 1.456 (1.056, 1.968) | 1.47 (1.089, 1.96) | 1.44 (1.046, 1.932) | 1.457 (1.097, 1.925) | 1.455 (1.061, 1.921) | 1.451 (1.082, 1.912) | 1.459 (1.102, 1.941) | 1.448 (1.077, 1.929) | 1.467 (1.072, 1.971) | 1.456 (1.056, 1.927) | 1.456 (1.056, 1.927) | 1.456 (1.056, 1.927) | 1.456 (1.05, 1.927) |
|  | None | Both | Males | 1.0 (1.0, 1.0) | 1.0 (1.0, 1.0) | 1.0 (1.0, 1.0) | 1.0 (1.0, 1.0) | 1.0 (1.0, 1.0) | 1.0 (1.0, 1.0) | 1.0 (1.0, 1.0) | 1.0 (1.0, 1.0) | 1.0 (1.0, 1.0) | 1.0 (1.0, 1.0) | 1.0 (1.0, 1.0) | 1.0 (1.0, 1.0) | 1.0 (1.0, 1.0) | 1.0 (1.0, 1.0) | 1.0 (1.0, 1.0) | 1.0 (1.0, 1.0) | 1.0 (1.0, 1.0) |
|  | None | Both | Females | 1.0 (1.0, 1.0) | 1.0 (1.0, 1.0) | 1.0 (1.0, 1.0) | 1.0 (1.0, 1.0) | 1.0 (1.0, 1.0) | 1.0 (1.0, 1.0) | 1.0 (1.0, 1.0) | 1.0 (1.0, 1.0) | 1.0 (1.0, 1.0) | 1.0 (1.0, 1.0) | 1.0 (1.0, 1.0) | 1.0 (1.0, 1.0) | 1.0 (1.0, 1.0) | 1.0 (1.0, 1.0) | 1.0 (1.0, 1.0) | 1.0 (1.0, 1.0) | 1.0 (1.0, 1.0) |

**Table S5.** Relative risks used by age and sex for each outcome for occupational exposure to noise and ergonomic factors

| **Risk - Outcome** | **Category** | **Morbidity/ Mortality** | **Sex** | **Age group** | | | | | | | | | | | | | | | | |
| --- | --- | --- | --- | --- | --- | --- | --- | --- | --- | --- | --- | --- | --- | --- | --- | --- | --- | --- | --- | --- |
|  |  |  |  | **15-19 years** | **20-24 years** | **25-29 years** | **30-34 years** | **35-39 years** | **40-44 years** | **45-49 years** | **50-54 years** | **55-59 years** | **60-64 years** | **65-69 years** | **70-74 years** | **75-79 years** | **80-84 years** | **85-89 years** | **90-94 years** | **95+ years** |
| **Occupational noise** | | | | | | | | | | | | | | | | | | | | |
| Mild hearing loss due to age-related and other hearing loss | High exposure, >90dB | Morbidity | Both | 7.477 (5.049, 11.13) | 7.423 (4.787, 10.95) | 5.504 (4.293, 6.843) | 5.456 (4.339, 6.828) | 3.07 (2.692, 3.51) | 3.081 (2.715, 3.498) | 2.546 (2.332, 2.774) | 2.554 (2.367, 2.752) | 1.849 (1.712, 1.995) | 1.85 (1.707, 1.995) | 1.45 (1.372, 1.526) | 1.454 (1.378, 1.529) | 1.131 (1.048, 1.211) | 1.131 (1.048, 1.211) | 1.131 (1.048, 1.211) | 1.131 (1.048, 1.211) | 1.131 (1.048, 1.211) |
| Mild hearing loss with ringing due to age-related and other hearing loss | High exposure, >90dB | Morbidity | Both | 7.477 (5.049, 11.13) | 7.423 (4.787, 10.95) | 5.504 (4.293, 6.843) | 5.456 (4.339, 6.828) | 3.07 (2.692, 3.51) | 3.081 (2.715, 3.498) | 2.546 (2.332, 2.774) | 2.554 (2.367, 2.752) | 1.849 (1.712, 1.995) | 1.85 (1.707, 1.995) | 1.45 (1.372, 1.526) | 1.454 (1.378, 1.529) | 1.131 (1.048, 1.211) | 1.131 (1.048, 1.211) | 1.131 (1.048, 1.211) | 1.131 (1.048, 1.211) | 1.131 (1.048, 1.211) |
| Moderate hearing loss due to age-related and other hearing loss | High exposure, >90dB | Morbidity | Both | 8.175 (4.62, 13.22) | 8.31 (4.828, 13.46) | 6.71 (4.785, 9.239) | 6.756 (4.705, 9.168) | 5.992 (4.34, 8.307) | 5.987 (4.304, 8.107) | 5.624 (3.954, 7.821) | 5.61 (3.96, 7.69) | 3.643 (2.576, 5.019) | 3.601 (2.494, 4.936) | 2.17 (1.597, 2.84) | 2.146 (1.656, 2.862) | 1.291 (1.06, 1.539) | 1.291 (1.06, 1.539) | 1.291 (1.06, 1.539) | 1.291 (1.06, 1.539) | 1.291 (1.06, 1.539) |
| Moderate hearing loss with ringing due to age-related and other hearing loss | High exposure, >90dB | Morbidity | Both | 8.175 (4.62, 13.22) | 8.31 (4.828, 13.46) | 6.71 (4.785, 9.239) | 6.756 (4.705, 9.168) | 5.992 (4.34, 8.307) | 5.987 (4.304, 8.107) | 5.624 (3.954, 7.821) | 5.61 (3.96, 7.69) | 3.643 (2.576, 5.019) | 3.601 (2.494, 4.936) | 2.17 (1.597, 2.84) | 2.146 (1.656, 2.862) | 1.291 (1.06, 1.539) | 1.291 (1.06, 1.539) | 1.291 (1.06, 1.539) | 1.291 (1.06, 1.539) | 1.291 (1.06, 1.539) |
| Moderately severe hearing loss due to age-related and other hearing loss | High exposure, >90dB | Morbidity | Both | 8.27 (4.807, 13.22) | 8.233 (4.709, 13.12) | 6.764 (4.814, 9.271) | 6.726 (4.762, 9.201) | 6.007 (4.114, 8.374) | 6.072 (4.34, 8.475) | 5.638 (3.943, 7.822) | 5.704 (4.007, 8.022) | 3.638 (2.576, 4.959) | 3.591 (2.583, 4.981) | 2.162 (1.64, 2.816) | 2.168 (1.631, 2.827) | 1.297 (1.06, 1.561) | 1.297 (1.06, 1.561) | 1.297 (1.06, 1.561) | 1.297 (1.06, 1.561) | 1.297 (1.06, 1.561) |
| Moderately severe hearing loss with ringing due to age-related and other hearing loss | High exposure, >90dB | Morbidity | Both | 8.27 (4.807, 13.22) | 8.233 (4.709, 13.12) | 6.764 (4.814, 9.271) | 6.726 (4.762, 9.201) | 6.007 (4.114, 8.374) | 6.072 (4.34, 8.475) | 5.638 (3.943, 7.822) | 5.704 (4.007, 8.022) | 3.638 (2.576, 4.959) | 3.591 (2.583, 4.981) | 2.162 (1.64, 2.816) | 2.168 (1.631, 2.827) | 1.297 (1.06, 1.561) | 1.297 (1.06, 1.561) | 1.297 (1.06, 1.561) | 1.297 (1.06, 1.561) | 1.297 (1.06, 1.561) |
| Severe hearing loss with ringing due to age-related and other hearing loss | High exposure, >90dB | Morbidity | Both | 8.249 (4.72, 13.18) | 8.292 (4.754, 13.144) | 6.707 (4.73, 9.34) | 6.687 (4.739, 9.181) | 6.075 (4.315, 8.372) | 5.983 (4.247, 8.221) | 5.66 (4.039, 7.875) | 5.61 (3.946, 7.534) | 3.62 (2.541, 5.01) | 3.583 (2.513, 5.117) | 2.138 (1.606, 2.802) | 2.173 (1.645, 2.807) | 1.294 (1.059, 1.569) | 1.294 (1.059, 1.569) | 1.294 (1.059, 1.569) | 1.294 (1.059, 1.569) | 1.294 (1.059, 1.569) |
| Severe hearing loss due to age-related and other hearing loss | High exposure, >90dB | Morbidity | Both | 8.249 (4.72, 13.18) | 8.292 (4.754, 13.144) | 6.707 (4.73, 9.34) | 6.687 (4.739, 9.181) | 6.075 (4.315, 8.372) | 5.983 (4.247, 8.221) | 5.66 (4.039, 7.875) | 5.61 (3.946, 7.534) | 3.62 (2.541, 5.01) | 3.583 (2.513, 5.117) | 2.138 (1.606, 2.802) | 2.173 (1.645, 2.807) | 1.294 (1.059, 1.569) | 1.294 (1.059, 1.569) | 1.294 (1.059, 1.569) | 1.294 (1.059, 1.569) | 1.294 (1.059, 1.569) |
| Profound hearing loss due to age-related and other hearing loss | High exposure, >90dB | Morbidity | Both | 8.33 (4.77, 13.62) | 8.437 (4.945, 13.539) | 6.69 (4.739, 9.26) | 6.734 (4.811, 9.238) | 5.974 (4.17, 8.13) | 5.981 (4.273, 8.413) | 5.629 (3.965, 7.879) | 5.589 (3.838, 7.693) | 3.591 (2.489, 5.087) | 3.607 (2.48, 5.048) | 2.144 (1.619, 2.83) | 2.173 (1.638, 2.822) | 1.294 (1.081, 1.562) | 1.294 (1.081, 1.562) | 1.294 (1.081, 1.562) | 1.294 (1.081, 1.562) | 1.294 (1.081, 1.562) |
| Profound hearing loss with ringing due to age-related and other hearing loss | High exposure, >90dB | Morbidity | Both | 8.33 (4.77, 13.62) | 8.437 (4.945, 13.539) | 6.69 (4.739, 9.26) | 6.734 (4.811, 9.238) | 5.974 (4.17, 8.13) | 5.981 (4.273, 8.413) | 5.629 (3.965, 7.879) | 5.589 (3.838, 7.693) | 3.591 (2.489, 5.087) | 3.607 (2.48, 5.048) | 2.144 (1.619, 2.83) | 2.173 (1.638, 2.822) | 1.294 (1.081, 1.562) | 1.294 (1.081, 1.562) | 1.294 (1.081, 1.562) | 1.294 (1.081, 1.562) | 1.294 (1.081, 1.562) |
| Complete hearing loss due to age-related and other hearing loss | High exposure, >90dB | Morbidity | Both | 8.242 (4.856, 13.285) | 8.371 (4.712, 13.317) | 6.771 (4.783, 9.41) | 6.703 (4.728, 9.419) | 5.932 (4.209, 8.003) | 5.941 (4.176, 8.222) | 5.62 (3.963, 7.916) | 5.628 (4.002, 7.742) | 3.625 (2.498, 5.132) | 3.625 (2.558, 5.036) | 2.17 (1.614, 2.802) | 2.17 (1.602, 2.881) | 1.291 (1.06, 1.548) | 1.291 (1.06, 1.548) | 1.291 (1.06, 1.548) | 1.291 (1.06, 1.548) | 1.291 (1.06, 1.548) |
| Complete hearing loss with ringing due to age-related and other hearing loss | High exposure, >90dB | Morbidity | Both | 8.242 (4.856, 13.285) | 8.371 (4.712, 13.317) | 6.771 (4.783, 9.41) | 6.703 (4.728, 9.419) | 5.932 (4.209, 8.003) | 5.941 (4.176, 8.222) | 5.62 (3.963, 7.916) | 5.628 (4.002, 7.742) | 3.625 (2.498, 5.132) | 3.625 (2.558, 5.036) | 2.17 (1.614, 2.802) | 2.17 (1.602, 2.881) | 1.291 (1.06, 1.548) | 1.291 (1.06, 1.548) | 1.291 (1.06, 1.548) | 1.291 (1.06, 1.548) | 1.291 (1.06, 1.548) |
| Mild hearing loss due to age-related and other hearing loss | Low exposure, 85-90dB | Morbidity | Both | 2.807 (1.872, 3.963) | 2.764 (1.846, 4.054) | 2.953 (2.342, 3.708) | 2.934 (2.322, 3.605) | 2.176 (1.917, 2.47) | 2.172 (1.929, 2.475) | 1.988 (1.831, 2.153) | 1.991 (1.826, 2.156) | 1.551 (1.432, 1.675) | 1.553 (1.432, 1.686) | 1.301 (1.231, 1.37) | 1.3 (1.233, 1.37) | 1.091 (1.017, 1.172) | 1.091 (1.017, 1.172) | 1.091 (1.017, 1.172) | 1.091 (1.017, 1.172) | 1.091 (1.017, 1.172) |
| Mild hearing loss with ringing due to age-related and other hearing loss | Low exposure, 85-90dB | Morbidity | Both | 2.807 (1.872, 3.963) | 2.764 (1.846, 4.054) | 2.953 (2.342, 3.708) | 2.934 (2.322, 3.605) | 2.176 (1.917, 2.47) | 2.172 (1.929, 2.475) | 1.988 (1.831, 2.153) | 1.991 (1.826, 2.156) | 1.551 (1.432, 1.675) | 1.553 (1.432, 1.686) | 1.301 (1.231, 1.37) | 1.3 (1.233, 1.37) | 1.091 (1.017, 1.172) | 1.091 (1.017, 1.172) | 1.091 (1.017, 1.172) | 1.091 (1.017, 1.172) | 1.091 (1.017, 1.172) |
| Moderate hearing loss due to age-related and other hearing loss | Low exposure, 85-90dB | Morbidity | Both | 3.072 (1.768, 4.925) | 3.037 (1.78, 4.813) | 3.471 (2.441, 4.807) | 3.454 (2.449, 4.718) | 3.888 (2.724, 5.326) | 3.855 (2.701, 5.4) | 3.93 (2.791, 5.397) | 3.925 (2.715, 5.487) | 2.719 (1.933, 3.726) | 2.689 (1.863, 3.673) | 1.815 (1.363, 2.404) | 1.817 (1.363, 2.435) | 1.228 (1.022, 1.468) | 1.228 (1.022, 1.468) | 1.228 (1.022, 1.468) | 1.228 (1.022, 1.468) | 1.228 (1.022, 1.468) |
| Moderate hearing loss with ringing due to age-related and other hearing loss | Low exposure, 85-90dB | Morbidity | Both | 3.072 (1.768, 4.925) | 3.037 (1.78, 4.813) | 3.471 (2.441, 4.807) | 3.454 (2.449, 4.718) | 3.888 (2.724, 5.326) | 3.855 (2.701, 5.4) | 3.93 (2.791, 5.397) | 3.925 (2.715, 5.487) | 2.719 (1.933, 3.726) | 2.689 (1.863, 3.673) | 1.815 (1.363, 2.404) | 1.817 (1.363, 2.435) | 1.228 (1.022, 1.468) | 1.228 (1.022, 1.468) | 1.228 (1.022, 1.468) | 1.228 (1.022, 1.468) | 1.228 (1.022, 1.468) |
| Moderately severe hearing loss due to age-related and other hearing loss | Low exposure, 85-90dB | Morbidity | Both | 3.043 (1.733, 5.008) | 3.008 (1.821, 4.959) | 3.472 (2.48, 4.825) | 3.45 (2.427, 4.851) | 3.84 (2.68, 5.285) | 3.809 (2.686, 5.308) | 3.952 (2.757, 5.596) | 3.931 (2.788, 5.437) | 2.732 (1.891, 3.782) | 2.709 (1.9, 3.742) | 1.837 (1.33, 2.433) | 1.807 (1.37, 2.364) | 1.219 (1.017, 1.455) | 1.219 (1.017, 1.455) | 1.219 (1.017, 1.455) | 1.219 (1.017, 1.455) | 1.219 (1.017, 1.455) |
| Moderately severe hearing loss with ringing due to age-related and other hearing loss | Low exposure, 85-90dB | Morbidity | Both | 3.043 (1.733, 5.008) | 3.008 (1.821, 4.959) | 3.472 (2.48, 4.825) | 3.45 (2.427, 4.851) | 3.84 (2.68, 5.285) | 3.809 (2.686, 5.308) | 3.952 (2.757, 5.596) | 3.931 (2.788, 5.437) | 2.732 (1.891, 3.782) | 2.709 (1.9, 3.742) | 1.837 (1.33, 2.433) | 1.807 (1.37, 2.364) | 1.219 (1.017, 1.455) | 1.219 (1.017, 1.455) | 1.219 (1.017, 1.455) | 1.219 (1.017, 1.455) | 1.219 (1.017, 1.455) |
| Severe hearing loss with ringing due to age-related and other hearing loss | Low exposure, 85-90dB | Morbidity | Both | 3.023 (1.794, 4.956) | 2.972 (1.758, 4.883) | 3.444 (2.425, 4.739) | 3.482 (2.463, 4.764) | 3.837 (2.732, 5.263) | 3.867 (2.71, 5.354) | 3.98 (2.746, 5.531) | 3.943 (2.754, 5.548) | 2.711 (1.904, 3.803) | 2.693 (1.929, 3.736) | 1.83 (1.387, 2.422) | 1.825 (1.347, 2.402) | 1.218 (1.0, 1.475) | 1.218 (1.0, 1.475) | 1.218 (1.0, 1.475) | 1.218 (1.0, 1.475) | 1.218 (1.0, 1.475) |
| Severe hearing loss due to age-related and other hearing loss | Low exposure, 85-90dB | Morbidity | Both | 3.023 (1.794, 4.956) | 2.972 (1.758, 4.883) | 3.444 (2.425, 4.739) | 3.482 (2.463, 4.764) | 3.837 (2.732, 5.263) | 3.867 (2.71, 5.354) | 3.98 (2.746, 5.531) | 3.943 (2.754, 5.548) | 2.711 (1.904, 3.803) | 2.693 (1.929, 3.736) | 1.83 (1.387, 2.422) | 1.825 (1.347, 2.402) | 1.218 (1.0, 1.475) | 1.218 (1.0, 1.475) | 1.218 (1.0, 1.475) | 1.218 (1.0, 1.475) | 1.218 (1.0, 1.475) |
| Profound hearing loss due to age-related and other hearing loss | Low exposure, 85-90dB | Morbidity | Both | 3.023 (1.718, 5.041) | 3.018 (1.749, 4.908) | 3.455 (2.448, 4.681) | 3.446 (2.457, 4.727) | 3.875 (2.755, 5.323) | 3.848 (2.721, 5.132) | 3.943 (2.697, 5.393) | 3.944 (2.769, 5.398) | 2.697 (1.935, 3.775) | 2.69 (1.888, 3.733) | 1.833 (1.374, 2.406) | 1.813 (1.34, 2.386) | 1.222 (0.998, 1.479) | 1.222 (0.998, 1.479) | 1.222 (0.998, 1.479) | 1.222 (0.998, 1.479) | 1.222 (0.998, 1.479) |
| Profound hearing loss with ringing due to age-related and other hearing loss | Low exposure, 85-90dB | Morbidity | Both | 3.023 (1.718, 5.041) | 3.018 (1.749, 4.908) | 3.455 (2.448, 4.681) | 3.446 (2.457, 4.727) | 3.875 (2.755, 5.323) | 3.848 (2.721, 5.132) | 3.943 (2.697, 5.393) | 3.944 (2.769, 5.398) | 2.697 (1.935, 3.775) | 2.69 (1.888, 3.733) | 1.833 (1.374, 2.406) | 1.813 (1.34, 2.386) | 1.222 (0.998, 1.479) | 1.222 (0.998, 1.479) | 1.222 (0.998, 1.479) | 1.222 (0.998, 1.479) | 1.222 (0.998, 1.479) |
| Complete hearing loss due to age-related and other hearing loss | Low exposure, 85-90dB | Morbidity | Both | 3.004 (1.775, 4.913) | 2.986 (1.804, 4.882) | 3.482 (2.473, 4.715) | 3.478 (2.449, 4.702) | 3.858 (2.702, 5.284) | 3.813 (2.745, 5.341) | 3.917 (2.805, 5.383) | 3.985 (2.826, 5.477) | 2.701 (1.874, 3.658) | 2.695 (1.885, 3.731) | 1.812 (1.376, 2.371) | 1.824 (1.359, 2.407) | 1.222 (1.015, 1.475) | 1.222 (1.015, 1.475) | 1.222 (1.015, 1.475) | 1.222 (1.015, 1.475) | 1.222 (1.015, 1.475) |
| Complete hearing loss with ringing due to age-related and other hearing loss | Low exposure, 85-90dB | Morbidity | Both | 3.004 (1.775, 4.913) | 2.986 (1.804, 4.882) | 3.482 (2.473, 4.715) | 3.478 (2.449, 4.702) | 3.858 (2.702, 5.284) | 3.813 (2.745, 5.341) | 3.917 (2.805, 5.383) | 3.985 (2.826, 5.477) | 2.701 (1.874, 3.658) | 2.695 (1.885, 3.731) | 1.812 (1.376, 2.371) | 1.824 (1.359, 2.407) | 1.222 (1.015, 1.475) | 1.222 (1.015, 1.475) | 1.222 (1.015, 1.475) | 1.222 (1.015, 1.475) | 1.222 (1.015, 1.475) |
| Mild hearing loss due to age-related and other hearing loss | No exposure | Morbidity | Both | 1.0 (1.0, 1.0) | 1.0 (1.0, 1.0) | 1.0 (1.0, 1.0) | 1.0 (1.0, 1.0) | 1.0 (1.0, 1.0) | 1.0 (1.0, 1.0) | 1.0 (1.0, 1.0) | 1.0 (1.0, 1.0) | 1.0 (1.0, 1.0) | 1.0 (1.0, 1.0) | 1.0 (1.0, 1.0) | 1.0 (1.0, 1.0) | 1.0 (1.0, 1.0) | 1.0 (1.0, 1.0) | 1.0 (1.0, 1.0) | 1.0 (1.0, 1.0) | 1.0 (1.0, 1.0) |
| Mild hearing loss with ringing due to age-related and other hearing loss | No exposure | Morbidity | Both | 1.0 (1.0, 1.0) | 1.0 (1.0, 1.0) | 1.0 (1.0, 1.0) | 1.0 (1.0, 1.0) | 1.0 (1.0, 1.0) | 1.0 (1.0, 1.0) | 1.0 (1.0, 1.0) | 1.0 (1.0, 1.0) | 1.0 (1.0, 1.0) | 1.0 (1.0, 1.0) | 1.0 (1.0, 1.0) | 1.0 (1.0, 1.0) | 1.0 (1.0, 1.0) | 1.0 (1.0, 1.0) | 1.0 (1.0, 1.0) | 1.0 (1.0, 1.0) | 1.0 (1.0, 1.0) |
| Moderate hearing loss due to age-related and other hearing loss | No exposure | Morbidity | Both | 1.0 (1.0, 1.0) | 1.0 (1.0, 1.0) | 1.0 (1.0, 1.0) | 1.0 (1.0, 1.0) | 1.0 (1.0, 1.0) | 1.0 (1.0, 1.0) | 1.0 (1.0, 1.0) | 1.0 (1.0, 1.0) | 1.0 (1.0, 1.0) | 1.0 (1.0, 1.0) | 1.0 (1.0, 1.0) | 1.0 (1.0, 1.0) | 1.0 (1.0, 1.0) | 1.0 (1.0, 1.0) | 1.0 (1.0, 1.0) | 1.0 (1.0, 1.0) | 1.0 (1.0, 1.0) |
| Moderate hearing loss with ringing due to age-related and other hearing loss | No exposure | Morbidity | Both | 1.0 (1.0, 1.0) | 1.0 (1.0, 1.0) | 1.0 (1.0, 1.0) | 1.0 (1.0, 1.0) | 1.0 (1.0, 1.0) | 1.0 (1.0, 1.0) | 1.0 (1.0, 1.0) | 1.0 (1.0, 1.0) | 1.0 (1.0, 1.0) | 1.0 (1.0, 1.0) | 1.0 (1.0, 1.0) | 1.0 (1.0, 1.0) | 1.0 (1.0, 1.0) | 1.0 (1.0, 1.0) | 1.0 (1.0, 1.0) | 1.0 (1.0, 1.0) | 1.0 (1.0, 1.0) |
| Moderately severe hearing loss due to age-related and other hearing loss | No exposure | Morbidity | Both | 1.0 (1.0, 1.0) | 1.0 (1.0, 1.0) | 1.0 (1.0, 1.0) | 1.0 (1.0, 1.0) | 1.0 (1.0, 1.0) | 1.0 (1.0, 1.0) | 1.0 (1.0, 1.0) | 1.0 (1.0, 1.0) | 1.0 (1.0, 1.0) | 1.0 (1.0, 1.0) | 1.0 (1.0, 1.0) | 1.0 (1.0, 1.0) | 1.0 (1.0, 1.0) | 1.0 (1.0, 1.0) | 1.0 (1.0, 1.0) | 1.0 (1.0, 1.0) | 1.0 (1.0, 1.0) |
| Moderately severe hearing loss with ringing due to age-related and other hearing loss | No exposure | Morbidity | Both | 1.0 (1.0, 1.0) | 1.0 (1.0, 1.0) | 1.0 (1.0, 1.0) | 1.0 (1.0, 1.0) | 1.0 (1.0, 1.0) | 1.0 (1.0, 1.0) | 1.0 (1.0, 1.0) | 1.0 (1.0, 1.0) | 1.0 (1.0, 1.0) | 1.0 (1.0, 1.0) | 1.0 (1.0, 1.0) | 1.0 (1.0, 1.0) | 1.0 (1.0, 1.0) | 1.0 (1.0, 1.0) | 1.0 (1.0, 1.0) | 1.0 (1.0, 1.0) | 1.0 (1.0, 1.0) |
| Severe hearing loss with ringing due to age-related and other hearing loss | No exposure | Morbidity | Both | 1.0 (1.0, 1.0) | 1.0 (1.0, 1.0) | 1.0 (1.0, 1.0) | 1.0 (1.0, 1.0) | 1.0 (1.0, 1.0) | 1.0 (1.0, 1.0) | 1.0 (1.0, 1.0) | 1.0 (1.0, 1.0) | 1.0 (1.0, 1.0) | 1.0 (1.0, 1.0) | 1.0 (1.0, 1.0) | 1.0 (1.0, 1.0) | 1.0 (1.0, 1.0) | 1.0 (1.0, 1.0) | 1.0 (1.0, 1.0) | 1.0 (1.0, 1.0) | 1.0 (1.0, 1.0) |
| Severe hearing loss due to age-related and other hearing loss | No exposure | Morbidity | Both | 1.0 (1.0, 1.0) | 1.0 (1.0, 1.0) | 1.0 (1.0, 1.0) | 1.0 (1.0, 1.0) | 1.0 (1.0, 1.0) | 1.0 (1.0, 1.0) | 1.0 (1.0, 1.0) | 1.0 (1.0, 1.0) | 1.0 (1.0, 1.0) | 1.0 (1.0, 1.0) | 1.0 (1.0, 1.0) | 1.0 (1.0, 1.0) | 1.0 (1.0, 1.0) | 1.0 (1.0, 1.0) | 1.0 (1.0, 1.0) | 1.0 (1.0, 1.0) | 1.0 (1.0, 1.0) |
| Profound hearing loss due to age-related and other hearing loss | No exposure | Morbidity | Both | 1.0 (1.0, 1.0) | 1.0 (1.0, 1.0) | 1.0 (1.0, 1.0) | 1.0 (1.0, 1.0) | 1.0 (1.0, 1.0) | 1.0 (1.0, 1.0) | 1.0 (1.0, 1.0) | 1.0 (1.0, 1.0) | 1.0 (1.0, 1.0) | 1.0 (1.0, 1.0) | 1.0 (1.0, 1.0) | 1.0 (1.0, 1.0) | 1.0 (1.0, 1.0) | 1.0 (1.0, 1.0) | 1.0 (1.0, 1.0) | 1.0 (1.0, 1.0) | 1.0 (1.0, 1.0) |
| Profound hearing loss with ringing due to age-related and other hearing loss | No exposure | Morbidity | Both | 1.0 (1.0, 1.0) | 1.0 (1.0, 1.0) | 1.0 (1.0, 1.0) | 1.0 (1.0, 1.0) | 1.0 (1.0, 1.0) | 1.0 (1.0, 1.0) | 1.0 (1.0, 1.0) | 1.0 (1.0, 1.0) | 1.0 (1.0, 1.0) | 1.0 (1.0, 1.0) | 1.0 (1.0, 1.0) | 1.0 (1.0, 1.0) | 1.0 (1.0, 1.0) | 1.0 (1.0, 1.0) | 1.0 (1.0, 1.0) | 1.0 (1.0, 1.0) | 1.0 (1.0, 1.0) |
| Complete hearing loss due to age-related and other hearing loss | No exposure | Morbidity | Both | 1.0 (1.0, 1.0) | 1.0 (1.0, 1.0) | 1.0 (1.0, 1.0) | 1.0 (1.0, 1.0) | 1.0 (1.0, 1.0) | 1.0 (1.0, 1.0) | 1.0 (1.0, 1.0) | 1.0 (1.0, 1.0) | 1.0 (1.0, 1.0) | 1.0 (1.0, 1.0) | 1.0 (1.0, 1.0) | 1.0 (1.0, 1.0) | 1.0 (1.0, 1.0) | 1.0 (1.0, 1.0) | 1.0 (1.0, 1.0) | 1.0 (1.0, 1.0) | 1.0 (1.0, 1.0) |
| Complete hearing loss with ringing due to age-related and other hearing loss | No exposure | Morbidity | Both | 1.0 (1.0, 1.0) | 1.0 (1.0, 1.0) | 1.0 (1.0, 1.0) | 1.0 (1.0, 1.0) | 1.0 (1.0, 1.0) | 1.0 (1.0, 1.0) | 1.0 (1.0, 1.0) | 1.0 (1.0, 1.0) | 1.0 (1.0, 1.0) | 1.0 (1.0, 1.0) | 1.0 (1.0, 1.0) | 1.0 (1.0, 1.0) | 1.0 (1.0, 1.0) | 1.0 (1.0, 1.0) | 1.0 (1.0, 1.0) | 1.0 (1.0, 1.0) | 1.0 (1.0, 1.0) |
| **Occupational ergonomic factors** | | | | | | | | | | | | | | | | | | | | |
| Low back pain | Professional, technical and related workers | Morbidity | Both | 1.173 (1.066, 1.282) | 1.172 (1.062, 1.283) | 1.169 (1.065, 1.283) | 1.17 (1.062, 1.284) | 1.17 (1.062, 1.285) | 1.172 (1.062, 1.283) | 1.171 (1.071, 1.27) | 1.169 (1.063, 1.288) | 1.171 (1.059, 1.281) | 1.17 (1.058, 1.286) | 1.17 (1.07, 1.279) | 1.172 (1.065, 1.287) | 1.172 (1.07, 1.283) | 1.172 (1.07, 1.283) |  |  |  |
|  | Administrative and managerial workers | Morbidity | Both | 1.211 (0.964, 1.508) | 1.21 (0.964, 1.492) | 1.209 (0.965, 1.487) | 1.209 (0.963, 1.524) | 1.207 (0.976, 1.496) | 1.207 (0.965, 1.5) | 1.205 (0.946, 1.489) | 1.205 (0.967, 1.472) | 1.205 (0.961, 1.509) | 1.203 (0.948, 1.515) | 1.209 (0.976, 1.479) | 1.21 (0.964, 1.49) | 1.203 (0.961, 1.501) | 1.203 (0.961, 1.501) |  |  |  |
|  | Clerical and related workers | Morbidity | Both | 1.0 (1.0, 1.0) | 1.0 (1.0, 1.0) | 1.0 (1.0, 1.0) | 1.0 (1.0, 1.0) | 1.0 (1.0, 1.0) | 1.0 (1.0, 1.0) | 1.0 (1.0, 1.0) | 1.0 (1.0, 1.0) | 1.0 (1.0, 1.0) | 1.0 (1.0, 1.0) | 1.0 (1.0, 1.0) | 1.0 (1.0, 1.0) | 1.0 (1.0, 1.0) | 1.0 (1.0, 1.0) |  |  |  |
|  | Sales workers | Morbidity | Both | 1.22 (1.029, 1.434) | 1.21 (1.018, 1.418) | 1.213 (1.028, 1.434) | 1.214 (1.004, 1.448) | 1.207 (1.017, 1.445) | 1.218 (1.016, 1.455) | 1.212 (1.012, 1.444) | 1.216 (1.01, 1.448) | 1.219 (1.019, 1.45) | 1.211 (1.014, 1.444) | 1.213 (1.015, 1.455) | 1.21 (1.007, 1.423) | 1.214 (1.017, 1.446) | 1.214 (1.017, 1.446) |  |  |  |
|  | Service workers | Morbidity | Both | 1.472 (1.385, 1.568) | 1.472 (1.383, 1.569) | 1.471 (1.372, 1.563) | 1.472 (1.382, 1.571) | 1.469 (1.378, 1.567) | 1.472 (1.377, 1.57) | 1.469 (1.375, 1.568) | 1.47 (1.378, 1.57) | 1.472 (1.379, 1.575) | 1.472 (1.381, 1.572) | 1.474 (1.386, 1.571) | 1.47 (1.377, 1.568) | 1.472 (1.38, 1.571) | 1.472 (1.38, 1.571) |  |  |  |
|  | Agriculture, animal husbandry and forestry workers, fishermen and hunters | Morbidity | Both | 3.789 (2.58, 5.376) | 3.762 (2.621, 5.284) | 3.869 (2.642, 5.486) | 3.775 (2.569, 5.369) | 3.774 (2.606, 5.314) | 3.771 (2.532, 5.317) | 3.793 (2.632, 5.361) | 3.785 (2.556, 5.333) | 3.776 (2.645, 5.157) | 3.792 (2.536, 5.421) | 3.802 (2.684, 5.428) | 3.746 (2.609, 5.175) | 3.77 (2.635, 5.151) | 3.77 (2.635, 5.151) |  |  |  |
|  | Production and related workers, transport equipment operators and laborers | Morbidity | Both | 1.543 (1.409, 1.679) | 1.54 (1.406, 1.676) | 1.542 (1.415, 1.677) | 1.543 (1.413, 1.695) | 1.542 (1.416, 1.685) | 1.543 (1.418, 1.685) | 1.541 (1.402, 1.684) | 1.542 (1.41, 1.684) | 1.541 (1.404, 1.683) | 1.54 (1.414, 1.677) | 1.54 (1.408, 1.683) | 1.538 (1.408, 1.673) | 1.541 (1.41, 1.677) | 1.541 (1.41, 1.677) |  |  |  |
|  | Background | Morbidity | Both | 1.0 (1.0, 1.0) | 1.0 (1.0, 1.0) | 1.0 (1.0, 1.0) | 1.0 (1.0, 1.0) | 1.0 (1.0, 1.0) | 1.0 (1.0, 1.0) | 1.0 (1.0, 1.0) | 1.0 (1.0, 1.0) | 1.0 (1.0, 1.0) | 1.0 (1.0, 1.0) | 1.0 (1.0, 1.0) | 1.0 (1.0, 1.0) | 1.0 (1.0, 1.0) | 1.0 (1.0, 1.0) |  |  |  |

**Table S6.** The PAF of DALYs attributable to all occupational risk factors by causes and sexes in 1990 and 2017 in China

| Causes | Both sexes | | Females | | Males | |
| --- | --- | --- | --- | --- | --- | --- |
|  | 1990 | 2017 | 1990 | 2017 | 1990 | 2017 |
| Age-related and other hearing loss | 0.222 | 0.214 | 0.214 | 0.209 | 0.230 | 0.219 |
| Animal contact | 0.250 | 0.154 | 0.225 | 0.142 | 0.260 | 0.161 |
| Asthma | 0.085 | 0.072 | 0.075 | 0.060 | 0.093 | 0.081 |
| Chronic obstructive pulmonary disease | 0.193 | 0.192 | 0.185 | 0.183 | 0.201 | 0.199 |
| Drowning | 0.066 | 0.071 | 0.058 | 0.055 | 0.070 | 0.078 |
| Exposure to mechanical forces | 0.301 | 0.174 | 0.255 | 0.153 | 0.314 | 0.181 |
| Falls | 0.296 | 0.148 | 0.236 | 0.120 | 0.325 | 0.162 |
| Fire, heat, and hot substances | 0.234 | 0.165 | 0.218 | 0.163 | 0.247 | 0.167 |
| Foreign body | 0.047 | 0.062 | 0.039 | 0.056 | 0.054 | 0.066 |
| Kidney cancer | 0.001 | 0.001 | 0.001 | 0.001 | 0.001 | 0.001 |
| Larynx cancer | 0.047 | 0.053 | 0.055 | 0.057 | 0.045 | 0.053 |
| Leukemia | 0.008 | 0.011 | 0.009 | 0.012 | 0.007 | 0.010 |
| Low back pain | 0.385 | 0.280 | 0.373 | 0.263 | 0.400 | 0.302 |
| Mesothelioma | 0.587 | 0.721 | 0.677 | 0.707 | 0.486 | 0.729 |
| Nasopharynx cancer | 0.011 | 0.010 | 0.012 | 0.011 | 0.010 | 0.010 |
| Other transport injuries | 0.347 | 0.180 | 0.349 | 0.174 | 0.345 | 0.184 |
| Other unintentional injuries | 0.329 | 0.183 | 0.272 | 0.150 | 0.347 | 0.189 |
| Ovarian cancer | 0.009 | 0.010 | 0.009 | 0.010 | - | - |
| Pneumoconiosis | 1.000 | 1.000 | 1.000 | 1.000 | 1.000 | 1.000 |
| Poisonings | 0.209 | 0.145 | 0.221 | 0.148 | 0.201 | 0.142 |
| Road injuries | 0.309 | 0.172 | 0.284 | 0.160 | 0.319 | 0.177 |
| Tracheal, bronchus, and lung cancer | 0.081 | 0.089 | 0.088 | 0.086 | 0.078 | 0.090 |

PAF: Population attributable fractions.
