# Supplementary material for: The disease burden attributable to 18 occupational risks in China: an analysis for the global burden of disease study 2017
Source: Environ Health. 2020 Feb 19;19:21. doi: 10.1186/s12940-020-00577-y (PMC7031932; doi:10.1186/s12940-020-00577-y)
Supplement: Supplementary file 2 — Additional file 2: Table S7. Attributable burden of all occupational risks by causes. Table S8. Sex-specific age-standardized SEVs of occupational risks. [file 12940_2020_577_MOESM2_ESM.docx]

**Table S7.** Attributable burden and 95% UIs of all occupational risks by causes in China, 2017

| Causes | Deaths | |  | DALYs | |
| --- | --- | --- | --- | --- | --- |
|  | Number | Age-standardized rate |  | Number (in thousand) | Age-standardized rate |
| **Chronic respiratory diseases** | |  |  |  |  |
| Chronic obstructive pulmonary disease | 185035(146456-225854) | 11.24(8.71-13.94) |  | 3913.1 (3305.2-4534.7) | 212.69(178.51-248.89) |
| Pneumoconiosis | 8901(8234-9589) | 0.48(0.44-0.51) |  | 247.6 (225.2-272.8) | 12.57(11.45-13.87) |
| Asthma | 792(631-992) | 0.04(0.03-0.05) |  | 114.8 (79.8-158.3) | 6.28(4.34-8.68) |
| **Cancer** |  |  |  |  |  |
| Tracheal, bronchus, and lung cancer | 57338(42389-74586) | 2.92(2.20-3.77) |  | 1352.9 (966.2-1781.8) | 65.46(47.17-85.74) |
| Mesothelioma | 2229(1979-2436) | 0.12(0.10-0.13) |  | 55.6 (48.4-61.8) | 2.71(2.36-3.02) |
| Leukemia | 549(279-801) | 0.03(0.02-0.04) |  | 25.2 (12.9-36.8) | 1.48(0.76-2.17) |
| Larynx cancer | 935(502-1546) | 0.05(0.03-0.08) |  | 24.9 (12.7-42.8) | 1.18(0.61-2.01) |
| Nasopharynx cancer | 211(144-304) | 0.01(0.01-0.01) |  | 8.2 (5.5-11.8) | 0.42(0.29-0.59) |
| Ovarian cancer | 367(177-609) | 0.02(0.01-0.03) |  | 7.1 (3.4-11.9) | 0.36(0.17-0.60) |
| Kidney cancer | 14(3-26) | 0.00(0.00-0.00) |  | 0.4 (0.1-0.8) | 0.02(0.00-0.04) |
| **Causes due to occupational injuries** |  |  |  |  |  |
| Road injuries | 38945(32699-46273) | 2.23(1.87-2.65) |  | 2320.6 (1915.1-2758.8) | 137.28(113.65-163.33) |
| Falls | 9435(6700-11822) | 0.51(0.37-0.64) |  | 876.2 (669.4-1115.4) | 50.13(38.36-63.85) |
| Exposure to mechanical forces | 4816(3602-5844) | 0.27(0.20-0.33) |  | 376.9 (294.2-477.0) | 21.87(17.10-27.59) |
| Drowning | 4478(3670-5365) | 0.27(0.22-0.33) |  | 222.2 (181.9-267.6) | 14.14(11.53-17.09) |
| Other unintentional injuries | 3388(2751-4153) | 0.20(0.16-0.24) |  | 194.9 (157.0-237.2) | 11.78(8.90-15.46) |
| Other transport injuries | 1510(1251-1825) | 0.09(0.07-0.10) |  | 198.4 (149.6-259.4) | 11.60(9.36-14.18) |
| Poisonings | 3089(1822-3923) | 0.18(0.10-0.22) |  | 152.0 (94.4-192.4) | 9.15(5.68-11.68) |
| Fire, heat, and hot substances | 810(632-1025) | 0.05(0.04-0.06) |  | 122.9 (84.7-176.5) | 7.20(4.95-10.33) |
| Foreign body | 713(585-864) | 0.04(0.03-0.05) |  | 66.9 (51.7-82.8) | 3.87(3.00-4.81) |
| Animal contact | 278(217-391) | 0.01(0.01-0.02) |  | 26.7 (19.5-36.1) | 1.55(1.13-2.10) |
| **Age-related and other hearing loss** | - | - |  | 1742.2 (1186.1-2455.4) | 90.92(61.88-128.17) |
| **Low back pain** | - | - |  | 2010.5 (1372.6-2791.7) | 107.48(74.45-147.55) |

UIs: uncertainty intervals; DALYs: disability-adjusted life year

**Table S8.** National sex-specific age-standardized SEVs of occupational risks between 1990 and 2017 in China

| Occupational risk factor | Females | | |  | Males | | |
| --- | --- | --- | --- | --- | --- | --- | --- |
|  | 1990 | 2017 | % change 1990-2017 |  | 1990 | 2017 | % change 1990-2017 |
| Asthmagens | 13.17(9.65-17.63) | 9.19(6.91-12.05) | -30.24(-45.07--13.16) |  | 15.09(11.71-19.12) | 12.32(9.88-15.32) | -18.37(-30.85--2.63) |
| Ergonomic factors | 21.63(19.16-24.05) | 13.88(11.50-16.61) | -35.83(-48.16--22.22) |  | 20.41(18.28-22.59) | 12.95(11.01-15.24) | -36.55(-46.44--24.60) |
| Arsenic | 0.40(0.17-0.69) | 0.45(0.19-0.77) | 10.70(-2.08-26.24) |  | 0.30(0.12-0.51) | 0.34(0.13-0.58) | 14.68(2.90-29.40) |
| Asbestos | 0.50(0.33-0.71) | 0.41(0.36-0.46) | -17.74(-46.7-28.78) |  | 0.35(0.26-0.50) | 0.62(0.53-0.69) | 75.18(17.29-132.08) |
| Benzene | 0.50(0.31-0.88) | 0.63(0.38-1.14) | 25.60(15.47-37.56) |  | 0.43(0.26-0.77) | 0.51(0.31-0.89) | 18.95(10.94-29.62) |
| Beryllium | 0.10(0.10-0.11) | 0.11(0.10-0.11) | 2.25(-2.81-7.51) |  | 0.08(0.08-0.08) | 0.08(0.08-0.09) | 0.37(-5.34-6.70) |
| Cadmium | 0.21(0.20-0.22) | 0.23(0.21-0.26) | 10.91(-1.22-24.67) |  | 0.16(0.15-0.16) | 0.18(0.16-0.20) | 14.30(3.42-27.69) |
| Chromium | 0.41(0.39-0.43) | 0.48(0.43-0.54) | 17.81(3.58-33.24) |  | 0.32(0.30-0.33) | 0.39(0.35-0.43) | 22.22(10.29-36.97) |
| Diesel engine exhaust | 1.50(1.43-1.57) | 1.68(1.54-1.82) | 12.18(1.84-23.09) |  | 1.54(1.45-1.62) | 1.81(1.68-1.95) | 17.60(7.49-27.95) |
| Formaldehyde | 0.81(0.76-0.85) | 0.94(0.81-1.07) | 16.47(0.30-33.95) |  | 0.65(0.61-0.69) | 0.80(0.71-0.89) | 22.17(8.21-39.42) |
| Nickel | 0.43(0.14-1.11) | 0.43(0.14-1.11) | 0.69(-10.29-14.44) |  | 0.35(0.11-0.91) | 0.35(0.11-0.92) | 2.10(-8.01-14.72) |
| PAHs | 0.83(0.79-0.87) | 0.96(0.86-1.07) | 16.05(2.49-30.53) |  | 0.64(0.61-0.67) | 0.77(0.71-0.85) | 20.27(8.81-34.18) |
| Silica | 3.66(1.92-8.08) | 3.30(1.69-7.40) | -10.00(-19.71-0.88) |  | 3.25(1.68-7.13) | 3.20(1.63-6.95) | -1.38(-10.92-10.02) |
| Sulfuric acid | 0.95(0.69-1.65) | 0.98(0.69-1.72) | 2.80(-7.36-13.49) |  | 0.70(0.50-1.21) | 0.77(0.54-1.36) | 9.96(0.40-21.00) |
| Trichloroethylene | 0.23(0.22-0.24) | 0.27(0.25-0.29) | 18.84(7.45-31.04) |  | 0.18(0.17-0.19) | 0.22(0.21-0.24) | 22.90(13.07-35.26) |
| Noise | 9.14(8.48-9.85) | 9.33(8.87-9.89) | 2.13(-2.18-7.06) |  | 9.36(8.70-10.10) | 9.17(8.67-9.75) | -2.01(-6.31-3.11) |
| PGFs | 9.29(7.13-12.40) | 9.34(7.27-12.38) | 0.48(-4.37-6.63) |  | 9.30(7.18-12.29) | 9.04(7.14-11.97) | -2.88(-7.79-2.98) |

SEV: summary exposure value; DEE: Diesel engine exhaust; PAHs: Polycyclic aromatic hydrocarbons; PGF: Particulate matter, gases, and fumes.
